# A hybrid approach to identifying and assessing interactions between climate action (SDG13) policies and a range of SDGs in a UK context

**DOI:** 10.1007/s43621-021-00051-w

**Published:** 2021-10-05

**Authors:** Samuel Stevenson, Alexandra Collins, Neil Jennings, Alexandre Koberle, Felix Laumann, Anthony A. Laverty, Paolo Vineis, Jeremy Woods, Ajay Gambhir

**Affiliations:** 1 Grantham Institute - Climate Change and the Environment, Imperial College London, Exhibition Road, London, SW7 2AZ UK; 2 Centre for Environmental Policy, Weeks Building, 16 - 18 Prince’s Gardens, London, SW7 1NE UK; 3 Department of Mathematics, Imperial College London, Weeks Building, 16 - 18 Prince’s Gardens, London, SW7 1NE UK; 4 School of Public Health, Imperial College London, Reynolds Building, St Dunstan’s Road, London, W6 8RP UK; 5 School of Public Health, Imperial College London, St Mary’s Hospital, Praed Street, London, W2 1NY UK; 6 Centre for Environmental Policy, Imperial College London, Weeks Building, 16 - 18 Prince’s Gardens, London, SW7 1NE UK

**Keywords:** Sustainable Development Goals, SDGs, Climate action, SDG interactions, Keyword search, Expert survey

## Abstract

In 2015 the United Nations drafted the Paris Agreement and established the Sustainable Development Goals (SDGs) for all nations. A question of increasing relevance is the extent to which the pursuit of climate action (SDG 13) interacts both positively and negatively with other SDGs. We tackle this question through a two-pronged approach: a novel, automated keyword search to identify linkages between SDGs and UK climate-relevant policies; and a detailed expert survey to evaluate these linkages through specific examples. We consider a particular subset of SDGs relating to health, economic growth, affordable and clean energy and sustainable cities and communities. Overall, we find that of the 89 UK climate-relevant policies assessed, most are particularly interlinked with the delivery of SDG 7 (Affordable and Clean Energy) and SDG 11 (Sustainable Cities and Communities) and that certain UK policies, like the Industrial Strategy and 25-Year Environment Plan, interlink with a wide range of SDGs. Focusing on these climate-relevant policies is therefore likely to deliver a wide range of synergies across SDGs 3 (Good Health and Well-being), 7, 8 (Decent Work and Economic Growth), 9 (Industry, Innovation and Infrastructure), 11, 14 (Life Below Water) and 15 (Life on Land). The expert survey demonstrates that in addition to the range of mostly synergistic interlinkages identified in the keyword search, there are also important potential trade-offs to consider. Our analysis provides an important new toolkit for the research and policy communities to consider interactions between SDGs, which can be employed across a range of national and international contexts.

**Supplementary Information:**

The online version contains supplementary material available at 10.1007/s43621-021-00051-w.

## Introduction

**Fig. 1:**
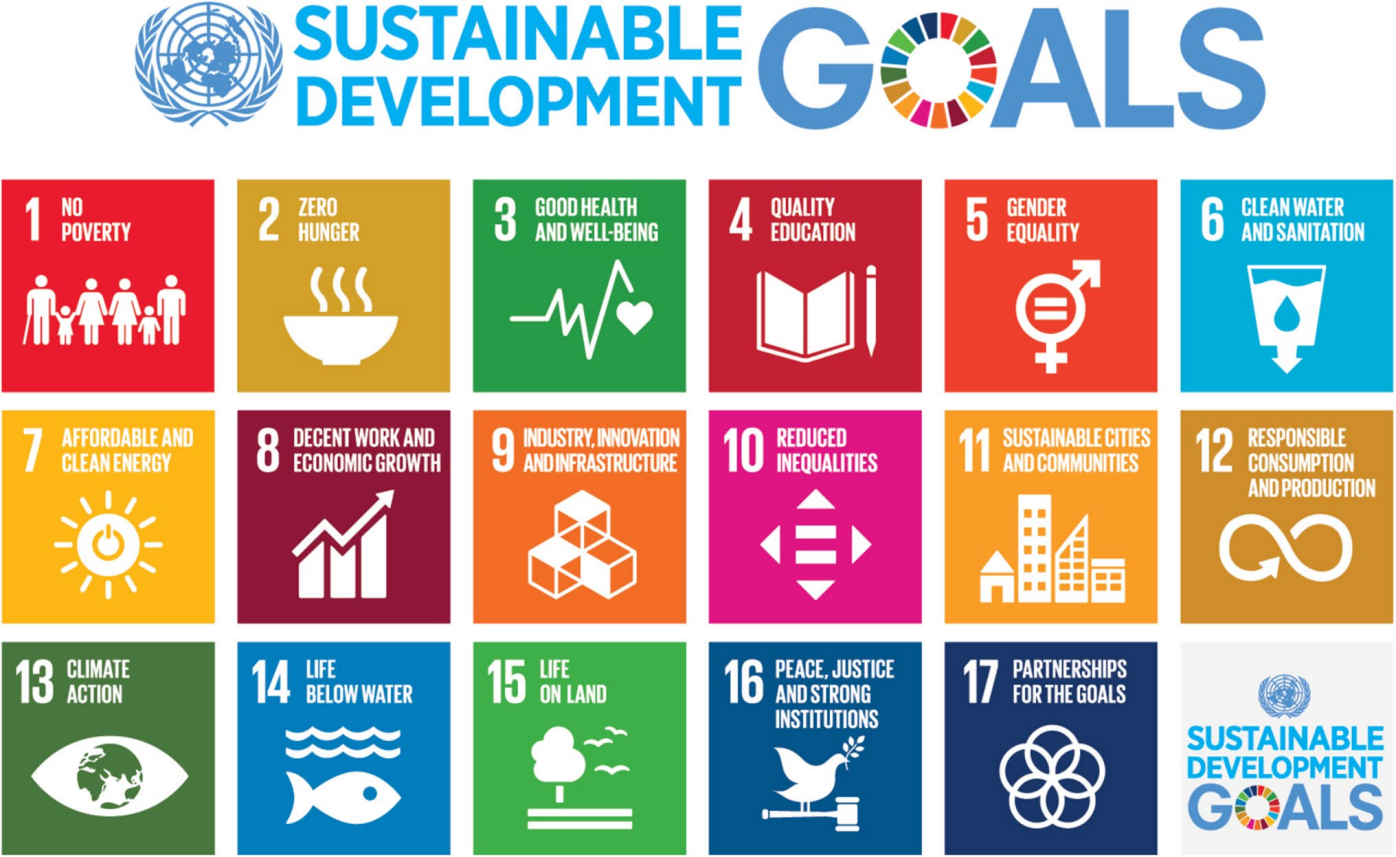
United Nations Sustainable Development Goals. (Source: https://sdgs.un.org/goals)

As the world approaches the 2030 Sustainable Development Goal (SDG) deadline, there is increasing scrutiny on how far we have come in achieving these goals since their inception in 2015. Equally, as the widespread impacts of climate change continue to unfold, there is growing concern that insufficient climate action is being taken in response. A key question is the extent to which climate action (SDG 13) interacts, both positively and negatively, with other SDGs (as shown in Fig. 1), such as the extent to which the pursuit of renewable energy policies impact on environmental or human health. Understanding these questions should allow policy makers to mitigate the trade-offs and amplify the synergies between taking climate action and delivering on the core objectives captured by the SDGs.

Significant questions also remain around how best to integrate these necessarily broad and internationally conceived goals into national policy frameworks and institutions. In an attempt to clarify these questions, we use the United Kingdom (UK) as a case study to test a hybrid methodology to identify SDG synergies and trade-offs, with a particular focus on climate action (SDG13). The UK is a suitable political context in which to test this relationship because it provides a case in which there is increasing urgency to respond to the SDGs in the context of a declared climate emergency. As well as being the first country to declare such an emergency, the UK is a leader on SDG 13 policies, establishing the Climate Change Act in 2008 [1], which was amended in 2019 to mandate net-zero emissions by 2050 [2]. The UK policy approach therefore provides fertile ground for the identification and evaluation of the extent to which SDG 13 interacts with other SDGs and how far these interactions can inform policy-making across the UK’s administrations.

Attempts to identify and understand SDG interactions are not new and the existing literature draws on a diverse range of methodological approaches, including: network analysis [3]; expert surveys [4]; system dynamics modelling [5]; natural language processing [6]; integrated assessment models [7, 8] and a range of statistical techniques [9–11]. Despite this, many of the methodologies remain onerous, time-consuming and, beyond a handful of examples, have not yet been deployed in tandem [12, 13]. In addition, whilst some studies have examined SDG interactions within regions or blocs [14, 15], limited work of this kind has been done at the single-nation level by examining large tranches of current national policy to contextualise identified SDG interactions. What is more, much of the existing literature has focused closely on interactions between SDGs *simpliciter*, rather than SDG-policy interactions, often by comparing goal-, target- or indicator-level objectives and data [16, 17]. A recent article from Bennich et al. has also highlighted the challenge of translating findings from this diverse range of methodologies into concrete policy actions and goals [18]. Specifically, the authors analysed a sample of 70 research papers from this field and discovered a need for studies which use SDG interactions analysis to directly inform policy making and innovation. Here we take up this challenge.

We take a subset of eight SDGs (3, 7, 8, 9, 11, 12, 14 and 15). This allows for a more detailed evaluation of interactions with goals which have particular relevance to both the UK and SDG 13. The exclusion of the eight remaining SDGs does not posit that these goals have no meaningful interactions with SDG 13, only that they were omitted from this study to keep the scope manageable whilst proving the basic concept. The principal objective of this work is to identify and assess interactions between SDG 13 and SDGs 3, 7, 8, 9, 11, 14 and 15 as they appear in UK climate-relevant policy documents. It is therefore not an exhaustive assessment of SDG 13 interactions more broadly, but a proof of concept exercise which applies a novel research methodology to those SDGs deemed relevant to the UK context.

The eight SDGs were selected by the research team as those most relevant to the UK case in particular (e.g. SDG 6 ‘Clean Water and Sanitation’ was not selected because the UK generally has high levels of potable water access and sanitation infrastructure). Focusing on the UK case allows for the development of a methodology which can rapidly identify and assess SDG interactions for use in both policy review and generation, for both the UK and for other countries. Combining a desk-based literature review on SDG interaction methodologies with a keyword search and expert survey provides a hybrid approach that mitigates the limitations of each method individually. Our approach clearly builds on existing work in this area, combining a semantic and expert survey dimension in order to identify and evaluate SDG interactions in a national policy context with a focus on SDG 13.

Recent work by the United Nations’ Department of Economic and Social Affairs, has developed an SDG tool which allows users to upload a policy document and extract semantic concepts (using keywords), linking them, and the document to various SDGs [19]. Unfortunately, at the time of undertaking this research, this tool was still in the developmental stages, and we recognise at least two main limitations: first, that documents can only be a maximum of 10,000 words which, in the UK case at least, excludes a significant number of longer, key strategy and policy documents e.g. Clean Growth Strategy or 25-Year Environment Plan. Second, whilst the tool extracts concepts by detecting the presence of keywords, it does not link these in any way to national departments. By contrast, our interest here is in understanding how SDG 13 interactions (synergies and trade-offs) with other SDGs map onto the UK and devolved administrations’ departments and policies. Therefore, here we outline an approach for a specially-designed SDG keyword search whose results can be used in both policy and research to map where synergies and trade-offs occur.

In summary, the rationale for our methodology design is three-fold: firstly, to develop a novel hybrid approach to rapidly identifying and assess SDG interactions; secondly, to identify and assess the co- benefits and trade-offs with other policy goals of taking climate action in the UK; and thirdly, to provide an analytical toolkit which can be applied by the research and policy community to a range of national and international settings.

The rest of this paper is structured as follows: “Materials and methods” section describes in detail the methods used; “Results” section presents the results of this study alongside analysis; “Discussion” section presents a discussion of the results and analysis and “Conclusions” section provides concluding comments.

## Materials and methods

**Fig. 2:**
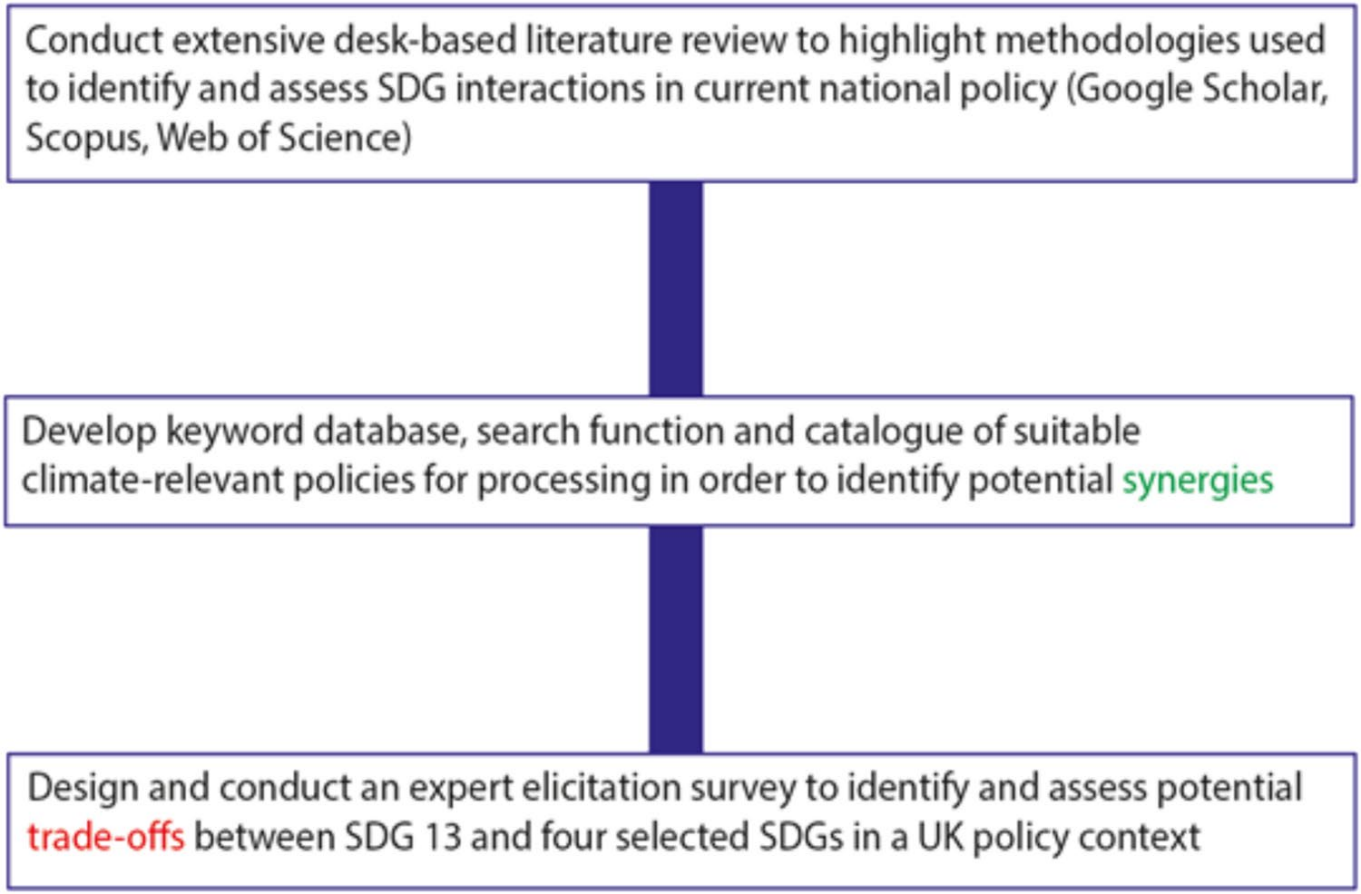
Flowchart outlining research methodology

This study was devised and conducted in 2020 over a nine-month period. The methodology for this research consisted of three parts, as shown in Fig. 2: a period of desk-based research; the development of an SDG-relevant keyword database and automated search; and a period of detailed expert survey. These are summarised in the sections below.

### Desk-based research

First, a desk-based literature review was undertaken with the objective of identifying the range of methods so far used to understand SDG interactions. Details of search combinations are shown in Supplementary Material 1 (S1). This phase indicated a number of different methods to determine interactions, including statistical methods [20], systems analysis [21] and integrated assessment models [8]. We deemed the use of keywords associated with SDGs and their sub-targets to be a promising method for identifying interactions for several reasons. First and foremost, it is simple and so can be conducted quickly, in contrast to many of the existing methods in this space. Second, there is limited research identifying and assessing SDG interactions at the national level using a language-based approach. Third, we wanted to test the hypothesis that semantic links between climate-relevant policies and SDGs can effectively identify potential co-benefits.

## Developing SDG keyword search and database

The second phase followed this approach by building an entirely new catalogue of SDG-relevant keywords to examine interactions. The catalogue was constructed by coding keywords from the UN’s description of the SDGs at both target- and goal-level (see S2). For example, keywords for SDG 7 (Affordable and Clean Energy) included ‘clean energy’, which forms part of the goal title, and ‘renewable energy’ which appears in the goal description. In addition, ‘indirect’ keywords were added. For example, SDG 12, target 12.4, calls for the “environmentally sound management of chemicals and wastes…[so as to] significantly reduce their release to air, water and soil…”, signifying the relevance of 'air/soil/water pollution/quality', as keywords for SDG 12. These keywords were then combined with an existing SDG glossary [22] to improve robustness and fill any gaps. The final list of keywords (see S2) was formed by combining these lists to create a composite database, further refined by optimising keywords for the search function (e.g. removal of duplicates, deletion of set phrases < 5 words). For further information on this process, see supplementary material S2.

This second phase also required the identification of relevant documents within which to search for keywords. Since our research question concerns itself with synergies and trade-offs between SDG 13 and other SDGs, we focused on identifying policy and strategy documents of direct relevance to SDG 13. We did this through carefully examining the UK’s Voluntary National Review (VNR), a mandatory national submission to the UN High-Level Political Forum covering the country’s progress towards the SDGs [23]. The UK VNR is divided into chapters, each discussing a different SDG and the UK policies and legislation related to its delivery. For the nine SDGs of interest in this project, each corresponding chapter was used to identify the relevant policy and legislation documents. All of the resulting 89 policies identified were included in this phase of the research, except those which obviously we deemed to have very limited, or no, relevance to SDG 13, (for example, the Tobacco Control Plan: 2017–2022). The remaining policies and legislation were then deemed relevant to SDG 13 either because they were referenced in the SDG 13 chapter of the VNR, or because of their explicit connection with, or relation to, climate action. For example, the Agriculture Bill (2019) isn’t included as a policy document in the SDG 13 chapter, but it has obvious linkages to climate action, given the large role of agriculture and land use in greenhouse gas emissions. As already noted, we focused on the interactions between SDG 13 and a subset of eight SDGs (3, 7, 8, 9, 11, 12, 14 and 15) from the full set of seventeen, in order to demonstrate a concept rather than provide a fully comprehensive mapping, whilst keeping the research tractable. Relevant policies were also included using the Welsh Supplementary VNR, whereas neither Scotland nor Northern Ireland has published a separate VNR supplement. For the full set of identified policies see S3.

Initial results from the keyword search highlighted two critical points: first, that matched keywords might not be contextually relevant—that is, they may appear in a context where positive interactions are not being discussed. We therefore undertook a manual context check to verify whether the matched keywords were identifying actual linkages rather than incidental mentions of keywords in a context not relevant to SDG interactions (e.g. ‘Energy’ in a reference to the ‘Department for Business Energy and Industrial Strategy’, which is merely the name of a UK Government Department rather than an indication of any meaningful interlinkage). The results from this context checking process showed that in 95% of cases, keyword matches indicated context-relevant linkages. Second, the results highlighted the highly synergistic (rather than trade-off) nature of interactions. This is to be expected given the nature of the documents, which are written by UK policy makers and thus unlikely to focus on challenging trade-offs associated with the selected policies. This did mean, however, that the keyword search was not well-suited to identifying negative interactions, and, as such, we designed a third phase of the research to identify trade-offs (as well as further synergies) through detailed expert survey, as discussed in the “Results” section below.

We use a Sankey diagram (see Fig. 4 in “Results” section) to illustrate the results of the keyword search, as this form of representation emphasises the most important contributors to an overall value or flow, enabling a clear identification of positively interacting SDGs. In order to demonstrate the relationship between climate-relevant policies, SDGs and UK departments, directorates and administrations, each of the policies collected for analysis was assigned the department responsible for its delivery (e.g. the “Road to Zero” policy document was assigned to the Department for Transport).

### Expert survey

Phase three built on phases one and two by using a survey alongside an approximately 60-minute interview, drawing on key stakeholders from across the UK central and devolved government to discuss potential trade-offs and synergies between SDGs in a UK context (see Table in S4). Respondents underwent a 60-minute session in which they completed a specially designed survey discussing both positive and negative interactions between our focal SDGs with a member of the research team. This survey compared SDGs at the target-level, in order to focus in on specific examples of potential synergies or trade-offs (see S5). The responses from phase two of the research are summarised in S6, and a selection of examples analysed in “Results” section of this paper.

Again, this aspect of our methodology was designed to focus on the identification and assessment of potential negative interactions (as well as identifying further positive interactions) between SDG 13 and other SDGs. Specifically, we took four of the original eight SDGs from the first part of our methodology (keyword search): SDG 3 (Good Health and Well-being); SDG 7 (Affordable and Clean Energy); SDG 8 (Decent Work and Economic Growth); and SDG 11 (Sustainable Cities and Communities). We focused on a subset rather than the full set to keep the research tractable and to ensure engagement amongst a large sample of experts. We chose this particular subset of SDGs because of the strength of their interaction with SDG 13 as identified in the keyword search, as well as because of their relevance to the UK’s current policy focus around national health systems in light of the Covid-19 pandemic, and possible policy responses to the lockdown-related recession.

**Fig. 3:**
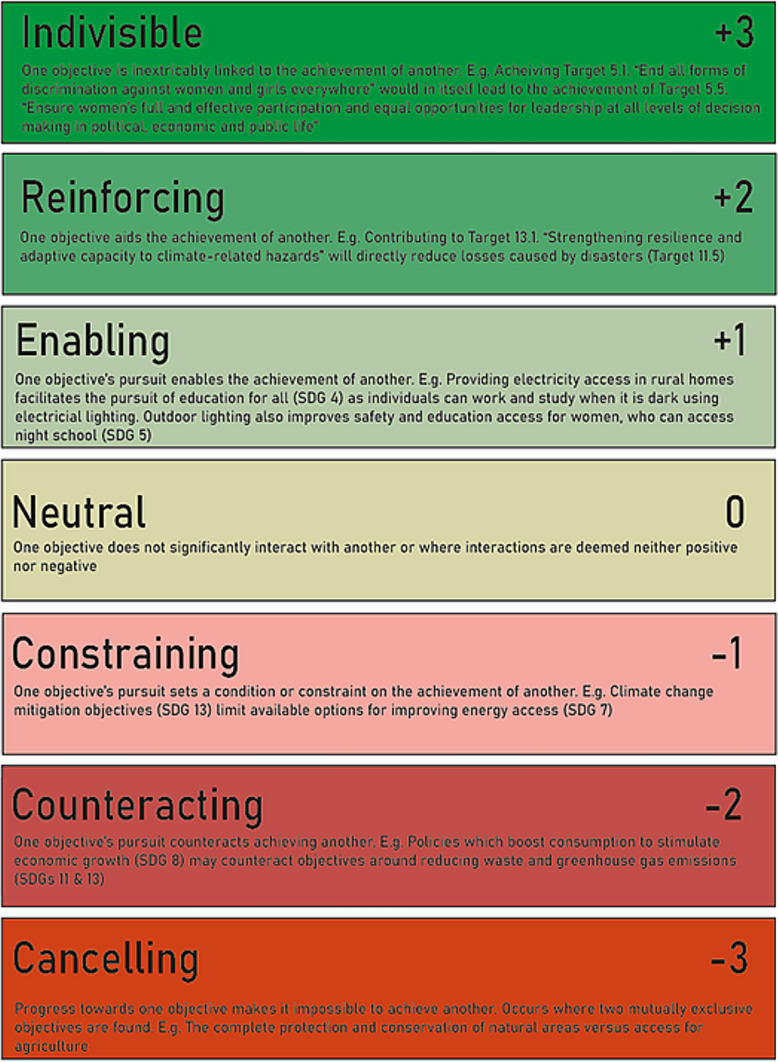
Nilsson scale for scoring interactions between the Sustainable Development Goals (adapted from McCollum et al. [12] with permission)

For each SDG of interest, we used the target-level descriptions relevant to the UK to generate example synergies and trade-offs, supported by identified examples from the academic literature. We then designed a survey which provided respondents with one example synergy and one example trade-off (where possible). Respondents were asked to score the strength of the interaction associated with the example synergy and trade-off given using the Nilsson scale (Fig. 3). They were then asked to generate any new example trade-offs or synergies associated with the same target-level descriptions and assign each new example with a Nilsson score.

The relevant SDG targets for UK climate action across which synergies and trade-offs were explored are as set out in Table 2. These targets were selected for three reasons. Firstly, for thematic reasons, because it is important to understand the relationship between climate action and public health, particularly given the current Covid-19 pandemic. Secondly, because the health co-benefits of climate action are likely to provide significant economic return [24–26]. Thirdly, for structural reasons, because SDG 13 targets 13.1, 13.2 and 13.3 and SDG 3 targets 3.3, 3.4, 3.6, 3.9 and 3.D are clearly relevant to UK climate action. By contrast, targets like 3.a, which is to ‘strengthen the WHO Framework Convention on Tobacco Control’, are not considered sufficiently relevant for the purposes of this study.

The Nilsson scale is a seven-point scoring system devised in 2016 [4]. The scale is designed to score interactions between SDGs and runs from − 3 to + 3, where − 3 is the strongest negative interaction score, 0 is neutral (no interaction) and + 3 is the strongest positive interaction score.

## Results

In this section, we present the results of our research in phases. “Identifying and assessing SDG 13 synergies: keyword search” section gives results for the keyword search and “Identifying and assessing SDG 13 trade-offs: expert survey” section describes results from the expert survey. “Identifying and assessing SDG 13 synergies: keyword search” section is broken down into subsections which show interactions between SDG 13 and SDGs 3 and 8, respectively. Each subsection of “Identifying and assessing SDG 13 synergies: keyword search” section has as its title the relevant SDG and sub-target (shown in brackets) whose interactions are presented.

### Identifying and assessing SDG 13 synergies: keyword search

The keyword search enabled the identification and assessment of SDGs with which climate-relevant policies are linked and, by analysing the specific matched words, an understanding of exactly how these policies are linked. To illustrate this, Table 1 below shows the top seven SDG 3 (Good Health and Well- being) keywords by frequency in the climate-relevant policies tested (shown in S3).

**Table 1:** Frequency of SDG 3 keywords in climate-relevant UK VNR policies

SDG 3 Keyword	Frequency
Health (‘healthy’/‘good’-/‘healthy lives’)	4289
Well-being (‘wellbeing’)	1142
Mental health	764
Air pollution	459
Disease(‘-s’)	403
Medical	131
Mortality (‘-rate’/‘-reducing’/‘premature-’/‘maternal-’/‘neonatal-’)	126
Healthcare	85

N.B. (‘’) = variation of keyword (‘- ’) = includes where this word follows keyword, and (‘ -‘) = where this word or letter follows keyword)

As well as the SDG 3 ‘title’ keywords (‘Good Health’ and ‘Well-being’), the climate-relevant policies frequently mention other important keywords such as ‘mental health’, ‘air pollution’ and ‘disease’, indicating the potential importance of these health-related themes in climate policy.

Crucially, there were only three *direct* references to the term “SDG 3” in the policy documents analysed, highlighting the importance of this keyword search in better understanding ‘hidden’ co-beneficial/thematic relationships between SDG 13-relevant policies and the other SDGs included in this analysis.

**Fig. 4:**
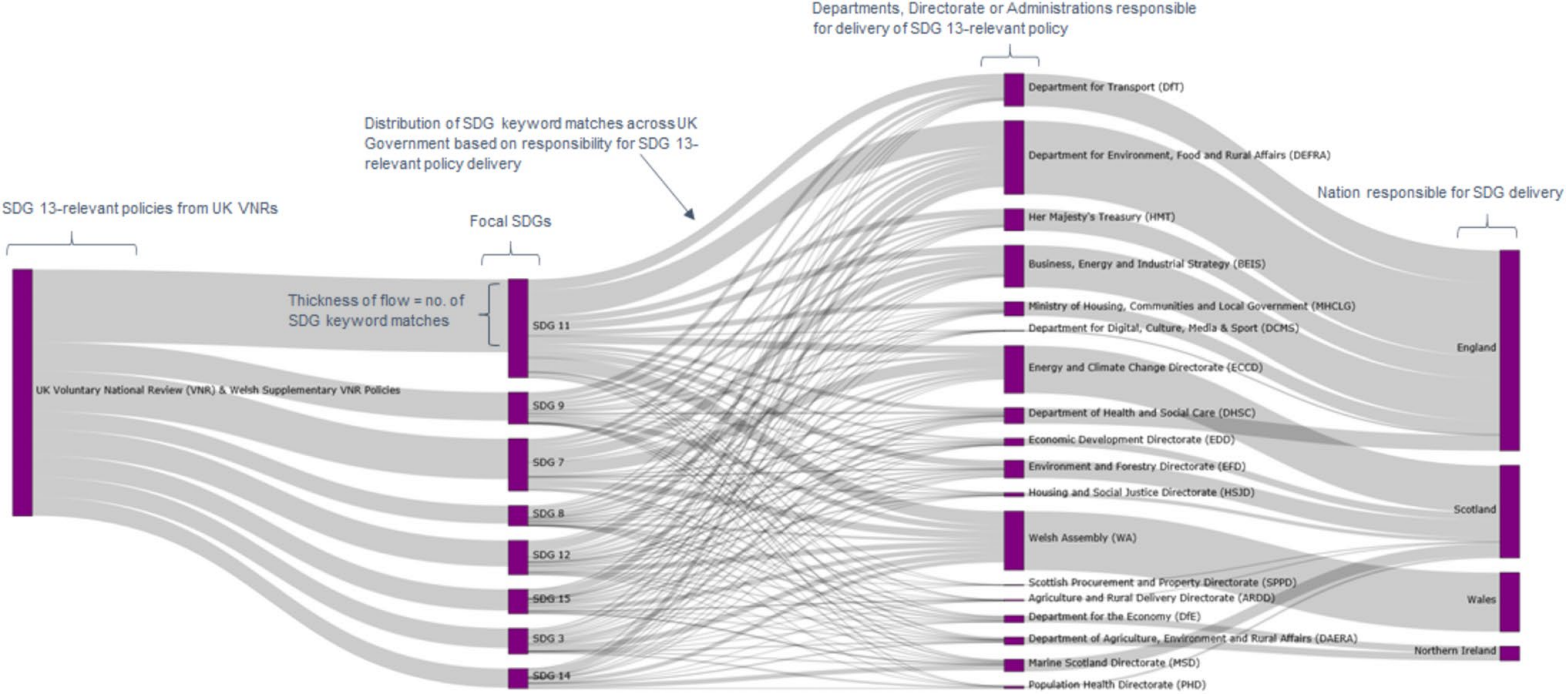
Sankey diagram showing the frequency of SDG-relevant keywords in climate action policies and their distribution across UK administrations, departments and directorates, as determined by the keyword search

Figure 4 illustrates the results of our keyword search—that is, the frequency of SDG-relevant keywords within climate-relevant policies from the UK VNRs. This enables an immediate view of the proportional SDG-relevance of a particular policy, as well as an indication of the most frequent SDG(s) across all climate policies tested. It shows that of the 89 climate-relevant policies to which the keyword search was applied, most are particularly interlinked with the delivery of SDG 7 (Affordable and Clean Energy) and SDG 11 (Sustainable Cities and Communities). This is shown in Fig. 4 by the fact that the lines leading into SDGs 7 and 11, which originate from the suite of SDG 13-relevant policy documents, are the thickest, and therefore have the greatest number of keywords relevant to these two SDGs.

Figure 4 also offers a more nuanced picture of the relationship between climate-relevant policies and SDG delivery across the UK’s departments, directorates and administrations. This was achieved by linking each policy to its administrating department during the policy collection phase. On this basis, in England, the Department for Environment, Food and Rural Affairs (DEFRA) appears to play an especially important role with regards to delivering SDG 11 (Sustainable Cities and Communities), as does the Department for Transport (DfT). This is intuitive for DfT given the central role of transport in cities, but less so for DEFRA, where the rural focus of the department makes its involvement in sustainability in cities notable. In both cases, this relationship is shown in Fig. 4 by the thickness of the lines from SDG 11 to the respective UK government departments, which signifies the distribution of SDG 11 keywords found in policies for which those departments are responsible.

### Identifying and assessing SDG 13 trade-offs: expert survey

The results from our expert survey are discussed in “SDG 13 (climate action) and SDG 3 (good health and well-being)” section and “SDG 13 (climate action) and SDG 8 (decent work and economic growth)” section below, focusing on interactions between SDG 13 and SDG 3 (Health), and SDG 13 and SDG 8 (Decent work and economic growth), respectively. Although interactions between SDG 13 and SDG 7 (Affordable and Clean Energy) and SDG 11 (Sustainable Cities and Communities) were also explored in the survey, these are not discussed in detail, for brevity. Results are shown, however, in S6. The analysis includes both responses to the examples provided by the survey and new examples generated by respondents. Although positive interactions are mentioned, we primarily focus our attention here on discussing potential negative SDG interactions, so as to supplement the (mostly positive) interaction analysis demonstrated in the keyword search phase. As well as the full set of responses being listed in S6, an excerpt from the survey is included as S5, showing the structure of the specialised SDG survey implemented in this study.

#### SDG 13 (Climate Action) and SDG 3 (Good Health and Well-being)

**Fig. 5:**
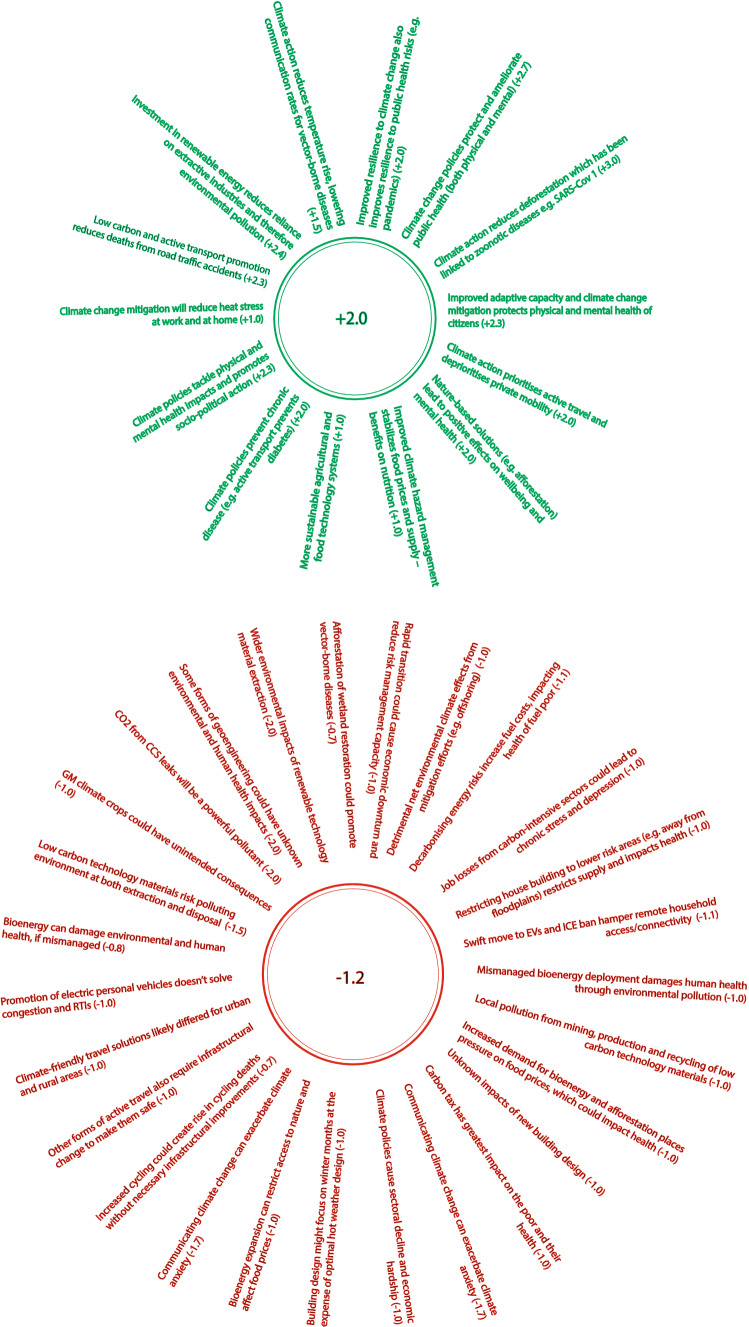
Summary of SDG 13–3 positive (green) and negative (red) interactions and Nilsson scores provided by expert survey phase. Central number indicates average score

On average, the scores awarded to the interactions between SDG 13 and SDG 3 were + **2.0** (for synergies) and **−** **1.2** (for trade-offs), as shown in Fig. 5. This average was produced using scores across all the example synergies and trade-offs (both pre-existing, as well as any newly identified by experts). Respondents generated a considerable number of new examples, with 8 new synergies and 22 new trade-offs.

The relevant SDG targets for UK climate action across which synergies and trade-offs were explored as set out in Table 2. These targets were selected for two reasons. Firstly, for thematic reasons, because it is important to understand the relationship between climate action and public health, particularly given the current Covid-19 pandemic. Secondly, for structural reasons, because for SDG 13, targets 13.1, 13.2 and 13.3 are directly relevant to UK climate action, as are SDG 3 targets 3.3, 3.4, 3.6, 3.9 and 3.D. By contrast, targets like 3.a, which is to ‘strengthen the WHO Framework Convention on Tobacco Control’, are not considered sufficiently relevant for the purposes of this study.

**Table 2:** Table showing targets from SDG 13 and SDG 3 relevant specifically to UK climate action

SDG Target	Description
13.1	Resilience and adaptive capacity to climate-related hazards
13.2	Integrate climate change measures into national policies, strategies and planning
13.3	Improve education, awareness-raising, and institutional capacity on climate change mitigation and adaptation
3.3	End epidemics and combat water-borne and other communicable diseases
3.4	Reduce premature mortality from non-communicable diseases and promote mental health and wellbeing
3.6	Halve the number of global deaths from road traffic accidents
3.9	Reduce the number of deaths and illness from hazardous chemicals and air, water and soil pollution
3.D	Strengthen capacity for early warning, risk reduction and management of national and global health risks

The following sub-sections discuss a selection of the elicited views on interactions between some of these targets.

##### Strengthen resilience and adaptive capacity to climate-related disasters (13.1), integrating climate change measures into national policy (13.2), reduce mortality from non-communicable diseases and promote mental health (3.4)

In this section, there was strong consensus amongst respondents on the example synergies, with an average Nilsson score of + **2** (reinforcing). Here, participants argued that strengthening the UK’s resilience to climate change by incorporating climate policy into the domestic policy approach would result in wide-reaching positive impacts for physical and mental health. Examples given include the impact of climate policies on transport, where the promotion of active travel modes such as walking or cycling alongside the curtailment of individual, fossil-fuelled private mobility will reduce non-communicable disease incidence rates (e.g. obesity, diabetes, cardiovascular diseases from sedentary lifestyles and respiratory diseases from poor ambient air quality). Equally, policies which discourage high levels of carbon intensive food consumption, such as a red meat, are also likely to reduce rates of cardiovascular disease [27].

With regards to trade-offs, respondents awarded the examples given in this section of the survey as **− 1.1** (constraining)**.** Overall, experts agreed that there is a material risk for the fuel poor as a result of decarbonising electricity and heat, due to the associated mental and physical health impacts of higher energy prices (e.g. cardiovascular and respiratory disease and anxiety).

Regarding electricity, experts noted that the UK has historically used mechanisms such as the Renewables Obligation (RO) and more recently, Contracts for Difference (CfD) to absorb part of the cost of large-scale power projects using consumer bills. Whilst the extent to which this cost can be absorbed by customers has been regulated by the Low Carbon Levy Control Framework (LCF) these mechanisms have resulted in higher electricity bills for consumers, thereby exacerbating fuel poverty. However, over time, as more renewable power projects have come online, the cost (£/MWh) of this electricity has also fallen [28]. Respondents argued that electricity costs are likely to continue falling and so this trade-off will diminish over time.

By contrast, experts highlighted that the decarbonisation of heat has gone largely unaddressed in the UK, and so the potential negative impacts will, in large part, be determined by future policy approaches. As a result, there is a risk that any move to absorb heat decarbonisation costs through consumer bills will increase heating costs and therefore the extent of fuel poverty. Overall, respondents considered this trade-off to be ‘constraining’, giving an average score of **− 1.1**, acknowledging that several supporting measures (such as improved energy efficiency) are likely to help offset future increases in heating fuel costs.

The second example trade-off provided concerns the potential mismanagement of bioenergy and the potentially negative impact on human health, for example via air and water pollution. This was almost exclusively considered a weak negative interaction (**− 1.1**), as the scope for significant negative impacts is very much tied to a number of factors including the type of bioenergy, location and scale of that bioenergy, and how it is both incentivised and regulated. Relatedly, there was additional concern around the associated land-use impacts of certain forms of bioenergy, e.g. biofuel production, and the impact this could have on human health through its interaction with arable land and therefore food price and availability, particularly as the UK begins to warm and the risk of pestilence, crop failure and drought increase.

Finally, there was concern from respondents that a potential trade-off between target 13.2 (integrate climate change measures into policy and planning) and 3.4 (reduce non-communicable disease mortality and promote mental health) could be the mental health (stress, anxiety, depression) as well as the subsequent and associated physical health impacts of unemployment in carbon intensive sectors. Scored at **− 1**, respondents argued that special attention must be given by policy makers to both the speed at which the net zero transition occurs and the suite of policies implemented. For example, policies which properly account for the risk of rapid job insecurity in fossil-related sectors and so invest adequately in concurrent re-skilling/re-training programmes could effectively support these communities and reduce the negative health (as well as socio-economic) impacts, ensuring a ‘just’ transition [29].

##### Growing awareness and institutional capacity on climate change mitigation and adaptation (13.2) and reduce mortality from non-communicable diseases and promote mental health (3.4)

In this section, there was strong consensus (**+ 2.2**) on the example synergy given: that improving awareness, education and institutional capacity on climate change mitigation, adaptation and impact reduction is likely to have an indirect benefit on human health and well-being. Most obviously, this is because improving our capacity for impact reduction will protect human life from the effects of climate change (e.g. flooding). Building on this, respondents also argued that as our institutional capacity for adaptation improves, policies which have a positive impact on human health and well-being will emerge—for example, incorporating proper ventilation and cooling into building design, which will reduce heat stress.

This section of the survey was presented with no-known trade-offs between targets 13.3 and 3.4. However, respondents argued that messaging around climate action is a very challenging communications task, and if mismanaged, can create panic, hysteria, and support the spread of misinformation. This is likely to have a material impact on the mental health and well-being of UK citizens, in the form of ‘climate anxiety’ and stress [30].

##### Strengthen resilience and adaptive capacity to climate-related disasters (13.1), integrating climate change measures into national policy (13.2), and halving global road traffic accidents (3.6)

Here, respondents agreed that there are strong synergies between climate action and reducing deaths from road traffic accidents. This is because policies which encourage low-carbon or active modes of travel, such as public transport, cycling or walking, remove individuals from private vehicles and therefore often from infrastructure which shares road traffic, reducing the accident risk. The average score for this synergy is + **2.2**.

On the other hand, the example trade-offs provided by respondents highlight that the decarbonisation of private mobility, e.g. promotion of electric vehicles may, at least initially, still present the same trade-offs around road traffic accidents (particularly as they are much quieter than ICE vehicles) and congestion, and because of this must be accompanied by equivalent investment in alternative low-carbon travel.

##### Strengthen resilience and adaptive capacity to climate-related disasters (13.1), integrating climate change measures into national policy (13.2) and reducing illness deaths from environmental pollution (3.9)

For this section, no new example synergies were generated by respondents beyond those given, which gained strong consensus (average score of + **2.4**)—strengthening resilience and response to climate change and reducing deaths from chemical, air, water and soil pollution/contamination.

In terms of trade-offs, the average score awarded for the example provided as well as the additional five examples was **−** **1.2**, indicating that various health-related considerations may constrain the objectives of 13.1 and 13.2. The example provided by the research team argues that bioenergy, if mismanaged, can affect environmental and therefore human health. Respondents built on this example by highlighting the wider potential health impacts of renewable technology supply chains (e.g. Li-Ion batteries, solar PV, electrolysers and CO_2_ leaks from Carbon Capture and Storage [CCS]), although, perhaps with the exception of CCS, noted that these are often outside of the UK. An additional point worth mentioning regards concern around future adaptation in sectors such as arable farming, where genetic modification decisions could impact human health in ways that are yet unknown (scored as −** 1.0**).

##### Strengthen resilience and adaptive capacity to climate-related disasters (13.1), integrating climate change measures into national policy (13.2), and strengthening management of national and global health risks (3.3)

In this final section comparing sub-targets of SDGs 13 and 3, an example synergy argues that there is a positive relationship between climate change mitigation and adaptation, and preparedness for large-scale public health risks. Overall, respondents scored this as a strong interaction with an average score of + **2.7**, citing the Covid-19 pandemic as an example of a large-scale public health risk. Specifically, many respondents highlighted that climate change mitigation and adaptation measures reduce the impact of climate-related events and therefore reduce strain on health infrastructure, better equipping the UK to deal with non-climate related public health crises. Equally, some noted the as-yet unclear links between Covid-19 lockdowns and pollution-related illnesses, where improving ambient air quality, for example, may also reduce both the morbidity and mortality rate of SARS-type viruses.

**Fig. 6:**
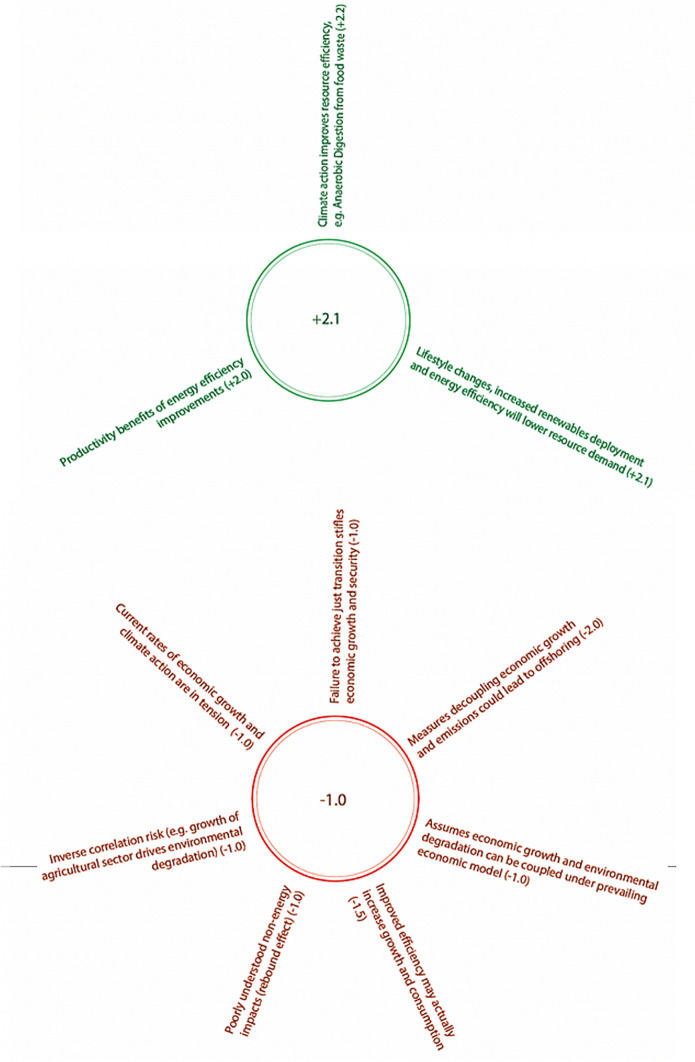
Summary of SDG 13–8 positive (green) and negative (red) interactions and Nilsson scores provided by expert survey phase. Central numbers indicate average score

In terms of trade-offs, respondents noted that a rapid climate transition without sufficient space for considered policy and decision-making processes could cause financial and economic disruption (e.g. rapid devaluation in fossil-related stocks and shares), which in turn could limit the UK government’s capacity to allocate funds on early warning and risk management processes across the economy (see Fig. 6).

#### SDG 13 (climate action) and SDG 8 (decent work and economic growth)

Overall, the average scores awarded to the interaction between SDG 13 and SDG 8 were + **2.3** (for synergies) and **−** **1.1** (for trade-offs). In this section, respondents also generated 1 new synergy and 8 new trade-offs, where no trade-offs were initially given in the survey.

Table 3 below shows the SDG targets considered most relevant for examining the relationship between climate action and SDG 8 (Decent Work and Economic Growth) in a UK context. We discuss these interactions further in just one sub-section “Integrating climate change measures into national policy (13.2) and improving global resource efficiency and reduced environmental degradation (8.4)”, as set out below.

**Table 3:** Table showing targets from SDG 13 and SDG 8 relevant specifically to UK climate action

SDG Target	Description
13.1	Resilience and adaptive capacity to climate-related hazards
13.2	Integrate climate change measures into national policies, strategies and planning
8.4	Improve global resource efficiency in consumption and production and decouple economic growth from environmental degradation

##### Integrating climate change measures into national policy (13.2) and improving global resource efficiency and reduced environmental degradation (8.4)

There was general consensus amongst experts that policies which promote climate action are likely to improve global resource efficiency in consumption and production, such as the use of processes which reduce food waste whilst generating low carbon heat and power (scored at + **2.2** overall). In addition, respondents recognised the importance of lifestyle changes supported by suitable climate action policy in improving global resource efficiency, whilst reducing environmental degradation (**+ 2.1**). The new example synergy generated argues that certain climate change mitigation measures, such as energy efficiency improvements, can also result in improved productivity (and therefore economic growth), scored as + **2.0** or ‘reinforcing’ on the Nilsson scale. In addition to the above there were also a number of potential trade- offs generated by respondents.

First, experts interviewed expressed concern that measures which effectively decoupled emissions and economic growth could simply lead to the offshoring of emissions elsewhere (**− 1.0**). It was argued that despite international emissions accounting frameworks this is still a very real risk in terms of domestic greenhouse emission reductions. According to experts interviewed, whilst this does not cancel the benefit of pursuing target 8.4, the risks of offshoring must be properly incorporated into policy assessment and design.

Secondly, others argued that whilst in principle targets 13.2 and 8.4 are synergistic, there are certain high carbon UK sectors that pose an inverse correlation risk, such as agriculture, where growth of the sector may actually drive environmental degradation (**-1.0**). Experts highlighted that although future technological developments promise to reduce the carbon intensity and environmental damage caused by operations within these sectors, there are questions around when these will reach technological readiness and the environmental impact of growth in high carbon sectors in the interim.

Thirdly, some respondents suggested that without some shift away from orthodox economic thinking, growth and environmental degradation cannot be decoupled. This potential trade-off was scored at **− 1.0**, indicating that whilst it is likely to constrain the achievement of targets 13.2 and 8.4, it is not considered counteracting or cancelling.

Finally, interviewees argued that there may be some very real but as-yet unknown non-energy impacts of improving resource efficiency through climate policies. For example, as energy efficiency improves people may consume more, resulting in negative impacts outside of energy (e.g. land use, water or wider environment).

## Discussion

These results, taken in their entirety, help demonstrate that our methodology greatly improves our understanding of how current climate action policy is distributed across the SDGs and UK departments, directorates, and administrations. This comes as a direct result of collecting and cataloguing policies for processing by the keyword search according to a target SDG or theme. We have shown that this first step alone can provide an informative picture of SDG delivery in the context of current UK climate policy, or indeed any policy framework screened relative to a target theme (e.g. education). Of course, as we have noted, this first step is also limited in its complexity in that it is based on the UK and Welsh Supplementary VNRs, documents which classify policies by SDG according to government thinking and may not do so exhaustively; thereby simplifying the relationship between these policies and the SDGs. Despite this, the cataloguing process is still an insightful initial mapping exercise for SDG policy analysis.

Furthermore, the results of the keyword search give a detailed indication of the SDG-relevance of climate action policy in the UK and a picture of how these policies (and therefore SDG-delivery) are distributed across UK departments, directorates and administrations. Our results suggest that even a relatively rudimentary keyword search, as used here, is a useful tool for indicating co-beneficial SDGs when assessing either groups of policies or individual policy documents. The picture presented in Fig. 4 is significantly more complex and interlinked than a consideration which only categorises policies in accordance with the VNR and does not apply any keyword search. Crucially, this approach can be modified to work at a number of scales—for example, at the city- or regional-level, simply by using policy or strategy documents which relate to the appropriate level of governance. We demonstrated a connection between the keyword search and expert survey phases by focusing on two of the highest scoring SDGs which were also contextually relevant to a post-Covid UK and the target SDG(s), (in our case SDG 13). Such an approach can be repeated for a variety of SDGs, making this a versatile, relatively rapid and low-cost approach to policy analysis and appraisal in light of SDG interactions. We recommend that a similar keyword search is implemented in the creation of a second UK VNR to map more comprehensively the interlinked nature of SDG delivery. More generally, we encourage the use of a keyword search in policy analysis so as to highlight hidden relationships between target policies, relevant SDGs and the departments, directorates and administrations responsible for their delivery.

The hybrid methodology to identify and evaluate SDG interactions is beneficial because it highlights interactions not evident in the VNRs. The keyword search has proven valuable in understanding the ‘SDG make-up’ of existing policies—for example, where policies are characterised as purely ‘energy’ policies, the keyword search has emphasised the extent to which they interact with the delivery of SDGs on health, sustainable cities and communities and economic growth. By highlighting the inter-SDG nature of policies, our approach can offer a more complete understanding of how the 2030 Agenda is being addressed at the national level, aiding both policy analysis and the design of new policies.

Our hybrid methodology enables cross-checking of the importance of specific inter-SDG links. For example, 50% of the SDG 13–SDG 3 interactions highlighted in the expert survey include ‘mental health’ and ‘disease’, which were also two of the highest-scoring keywords from the search. Experts were not shown the breakdown of highest-ranking keywords from the keyword search, so this independently shows that these themes are important positive interactions between SDGs 13 and 3, and that the keyword search is likely a useful tool for initially identifying significant themes. Moreover, the averaged Nilsson scores awarded to example synergies by our experts track the top-scoring SDGs from the keyword search, where SDG 11 scored + 2.4, SDG 7 scored + 2.3, SDG 8 scored + 2.1, and SDG 3 scored + 2.0. This suggests that the frequency of SDG-relevant keywords in climate-related policies highlighted by the keyword search may well be a useful initial indicator of how synergistic the policies are, which can then be substantiated by the expert survey.

The results of the keyword search (Fig. 4) indicate at least three policy implications. The first is that SDGs connected to SDG 13-relevant policies from the UK’s VNR are much more inter-departmental than the UK Government's classification would suggest. It may be that the UK Government has considered this elsewhere, outside of the VNR, but VNRs are designed to evidence in detail a country’s efforts towards achieving the SDGs and be representative of how a government classifies its policies in relation to these goals. Second, such results allow for a much more targeted approach to the inter-departmental coordination of SDG delivery, highlighting ‘hidden’ relationships between SDGs and departments. Third, as well as working for existing policy, this approach can be applied to newly devised policy to see if any key departments have been missed.

The keyword search is a valuable tool for several reasons. Perhaps most obviously, it allows us to link both a suite of policies as well as individual policies to SDGs which they might not otherwise be associated with on the basis of SDG-relevant keyword frequency within those documents. This gives a sense of the language-based interactions and so potential co-benefits (synergies) between current UK climate-relevant policies and our SDGs of interest. Second, when mapped to the departments, directorates, and administrations, responsible for the delivery of these policies, as in Fig. 4, it reframes (and updates) the current picture of SDG-delivery across the UK. Third, by looking at the most frequent keywords for an SDG of interest, we can better understand exactly *how* a certain policy, or group of policies, is linked to our SDGs—for example, that there is particular mention of ‘mental health’ in climate- relevant policies. This invites the possibility of (and we argue here, need for) comparative analysis, both inter-administrative (England–Scotland or England–Austria) and inter-temporal (policies from year X or first VNR versus second).

The results of this study both support and build on the existing SDG interaction literature. For example, Van Soest et al. indicated that current thinking on interactions, as understood through Integrated Assessment Models (IAMs), is particularly well suited to capturing the relationship between SDGs 13 and 7, for example [8]. Whilst our two-pronged approach also confirmed interactions between these SDGs in a UK context, it highlighted less obvious interactions, such as between SDGs 13 and 11, or 13 and 3. This speaks directly to Van Soest et al.’s work, which posits that methodologies for identifying interactions should draw on greater geographical detail (in our case, national-level policy), in order to provide more insight on potential, contextually relevant positive and negative SDG interactions [8]. Elsewhere, the potential synergies uncovered between climate action, good health and well-being and decent work and economic growth in the UK, push back on findings from Scherer et al., which suggest that the social and environmental SDGs are often in opposition [17]. As such, our results indicate that this methodology has produced novel and detailed outputs which contribute materially to the existing literature on SDG Interactions.

Fundamentally, our approach here builds on attempts to assess SDG interactions at a global scale [9], and operationalises the notion that interactions are heavily contingent on both geographical and temporal context [31]. As the first study applying this novel methodology to a national-level policy context, we demonstrate the capacity for rapidly identifying and assessing SDG integrations in a simple and replicable way, whilst also noting some of the approach’s limitations, including the ‘non-linear constraint’ element discussed by Laumann et al. [11]. Our conclusion highlights ways in which to mitigate these limitations in order to provide a useful methodology for initially mapping possible interactions between a given target SDG and some, or all, of the remaining SDGs.

Finally, the novel methodological approach taken here builds on the most recent recommendations from a bloc-level approach to SDG interaction assessment, proposed by Miola et al. [15]. Whilst our study looks at the UK, rather than EU context, we argue that the use of national- rather than bloc-level policy allows for greater granularity across both the keyword search and expert survey. This is because policy documents pertain only to the specific country in question, as do the remit of experts interviewed, allowing for a closer assessment of potential SDG interactions. Supporting the above recommendations that methodologies should provide significant levels of geographical detail (and therefore context), we argue that our approach should be replicated at the national (and perhaps even sub-national) level before being aggregated to assess commonalities in SDG interactions at larger scales (e.g. supra-national).

It is worth noting the limitations of using this particular hybrid methodology to investigate SDG interactions. The first is that, as with any keyword search, the quality of the outputs is determined by the quality of the inputs (policy documents and keywords within them). Policy documents must be “pre-treated” to remove any text which could produce false positives, such as footnotes and in-text referencing. A database of keywords must also be meaningful and tested to minimise false positives. As described in “Materials and methods” section (Methods), several steps were taken to minimise the possibility of false positives and to improve the robustness of the keyword search.

Second to note is that, although the presence of keywords may indicate positive interactions, the absence of these keywords does not necessarily indicate a trade-off. Again, because the language used in policy and strategy documents is skewed towards describing the benefits of such policy and rarely explores potentially negative impacts, a separate methodology is needed to identify and evaluate trade-offs. Our expert survey, whilst going some way to doing this, was not comprehensive across all of our focal SDGs.

Third, the keyword search, as applied in this study, is not well-equipped to deal with the temporal element of SDG interactions. It is well understood that the SDGs represent a complex system in which the relationship between objectives changes over time, often in a non-linear fashion. There is potential to develop the application of this keyword search so as to identify the presence of SDG-relevant keywords over time, either by comparing a group of policies from a particular date range to those from another date range, or by comparing the entire suite of selected policies (i.e. an inter-VNR assessment). This will offer some view (albeit simplistic) of the changing presence of synergistic topics within climate-relevant policies. It is also worth noting that the experts interviewed were highly cognisant of the temporal nature of SDG interactions. Respondents frequently presented the argument that interaction X is likely to diminish or augment over time, due to factors Y and Z. Whilst this understanding is far simpler than some of the existing mathematical work on non-linear SDG interactions [32] the consideration of how SDG interactions are likely to shift over time here is nevertheless critical.

Fourth, we should also clarify that the results from the keyword search in isolation indicate language-based interactions between climate-relevant policy and SDGs and are not therefore necessarily representative of real-world interactions (for this, we must elicit evaluation from experts). The keyword search is a heuristic tool which can guide decision making by highlighting the extent to which certain policies in the way they have been written and the language used to describe them link to that of the SDGs. This process highlights connections between climate-relevant policies and our eight SDGs that are otherwise not explicit, either because of the way they have been characterised thus far by government (e.g. in organigrammes of SDG delivery across departments or Voluntary National Reviews); or due to the purported aims of that policy—for example, ‘energy’ policy that may have obvious connections to SDG 7 (Affordable and Clean Energy) but also non-obvious connections to SDGs on poverty, health, and economic growth. However, as already noted, a comprehensive expert survey would help to substantiate and add important nuance to these identified interactions.

Finally, in addition to the above, it is important to reflect on the limitations of our focus on SDG 13 itself. We have taken climate-relevant policy and strategy to represent UK climate action, documents which Laumann et al. have classified as ‘input measures’ [11]. However, it is unclear what impact these policies, frameworks and strategies have on actually reducing the UK’s greenhouse gas emissions, as global mean temperature is not included as an indicator for SDG 13. We recommend here that in order to fully understand the relationship between the SDGs and climate change, we must include as a foundational indicator global mean temperature.

## Conclusions

By designing and implementing a hybrid methodology comprised of a keyword search combined with expert survey, we have developed an approach which can rapidly identify and assess SDG interactions, both synergies and trade-offs, in a national policy context. We have demonstrated the efficacy of this approach in a UK context, through examining interactions between SDG 13 (Climate Action), represented by climate-relevant policies from the UK VNRs, with a subset of eight other SDGs. Furthermore, we identify specific UK government departments and bodies that have strong interactions with particular SDGs, indicating the utility of this type of analysis for designing SDG policy delivery governance structures.

The complementary approach benefits from, on the one hand, the rapidity of the keyword search, and on the other hand, the nuance of the expert survey, which helps to both substantiate the keyword search and, more crucially, identify trade-offs which are unlikely to emerge from the keyword search alone. Even a relatively small sample size of experts (in this case 17) provided a rich set of potential negative (and positive) interactions to incorporate into the decision and policy-making process and signals the benefits of applying this same methodology to other scaled contexts. As such, we are confident that this methodology should be replicated and refined at a number of governance scales in order to expedite the process of both SDG policy analysis and policy generation.

It is hoped that this will improve our understanding of the relationship between these broad, internationally conceived goals and their application in real world national policy contexts today. Finally, we hope that the tools highlighted by this study are capable of improving our understanding of SDG delivery alongside large-scale societal challenges, such as climate change and the coronavirus pandemic.

## Supplementary Information

Additional file1 (PNG 1661 KB)

Additional file2 (PNG 736 KB)

Additional file3 (PNG 433 KB)

Additional file4 (PNG 395 KB)

Additional file5 (PNG 1587 KB)

Additional file6 (PDF 27755 KB)

## Data Availability

The datasets generated during and/or analysed during the current study are available from the corresponding author on reasonable request.
