# Supplementary figures and images for: A hybrid approach to identifying and assessing interactions between climate action (SDG13) policies and a range of SDGs in a UK context
Source: Discov Sustain. 2021 Oct 5;2(1):43. doi: 10.1007/s43621-021-00051-w (PMC8491187; doi:10.1007/s43621-021-00051-w)

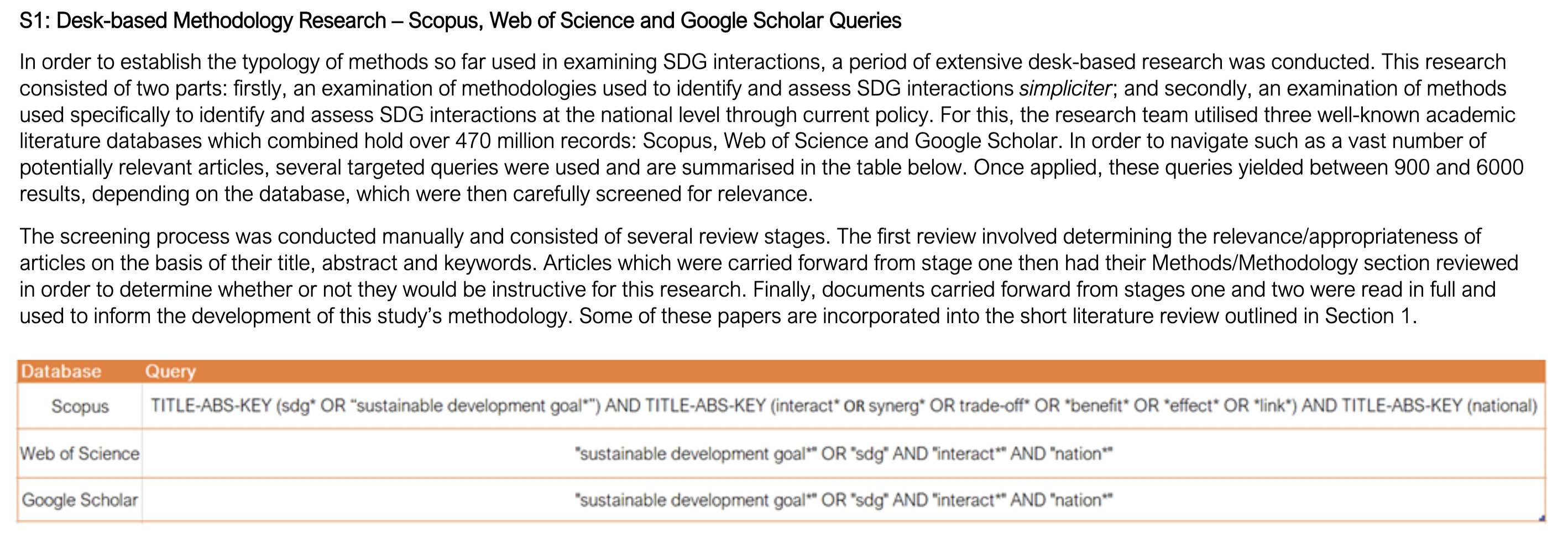

Supplement: Supplementary file 1 — Additional file1 (PNG 1661 KB) [file 43621_2021_51_MOESM1_ESM.png]

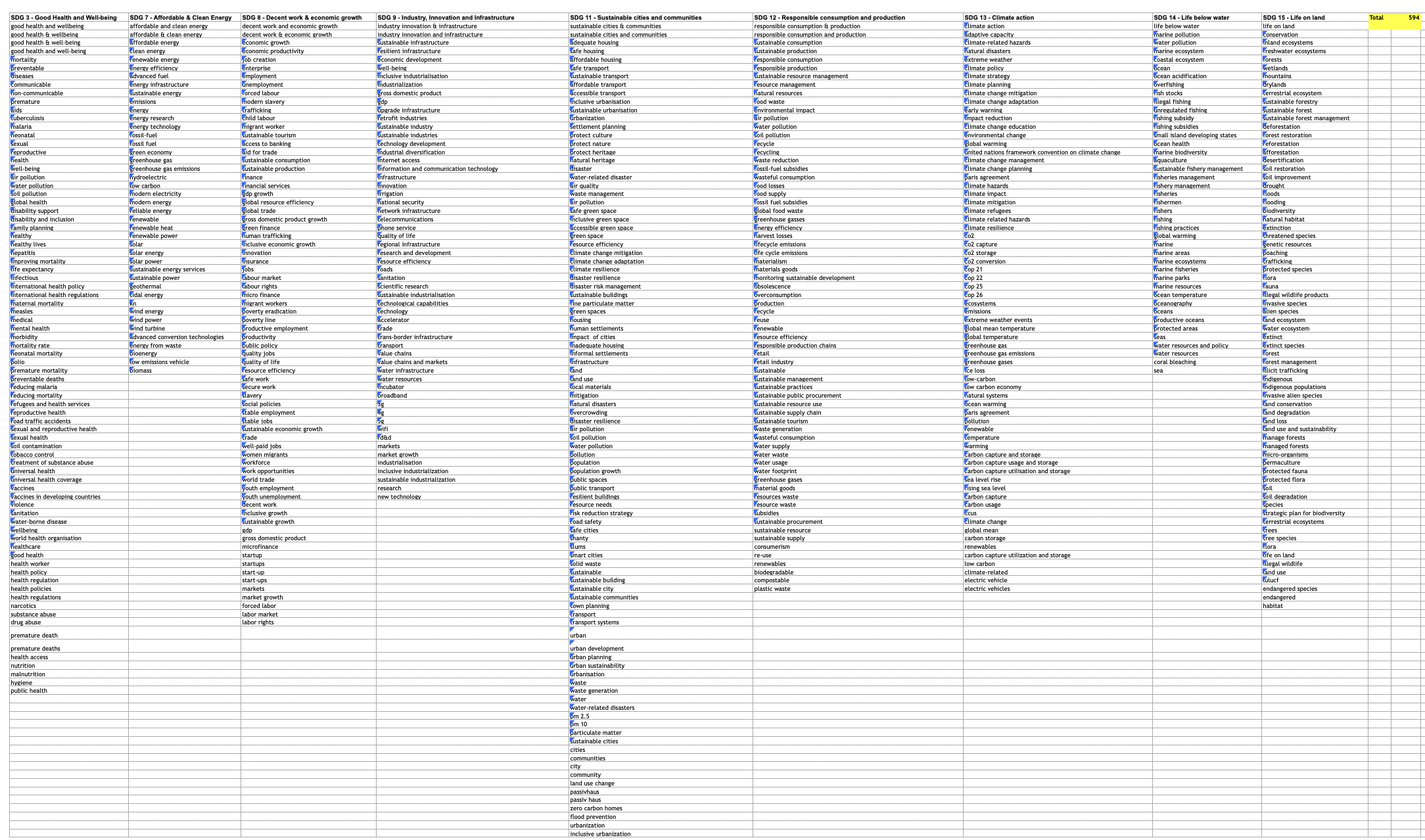

Supplement: Supplementary file 2 — Additional file2 (PNG 736 KB) [file 43621_2021_51_MOESM2_ESM.png]

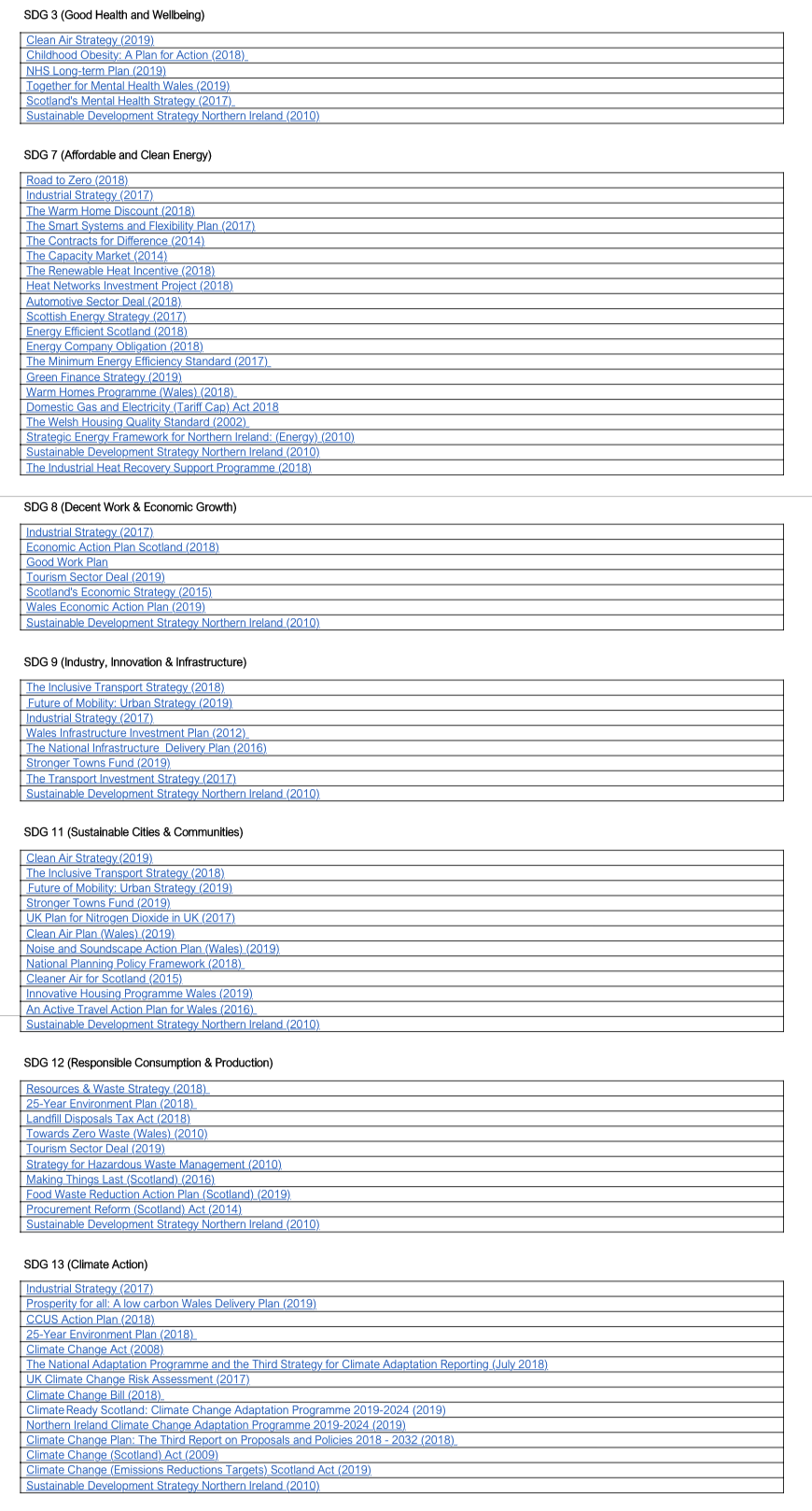

Supplement: Supplementary file 3 — Additional file3 (PNG 433 KB) [file 43621_2021_51_MOESM3_ESM.png]

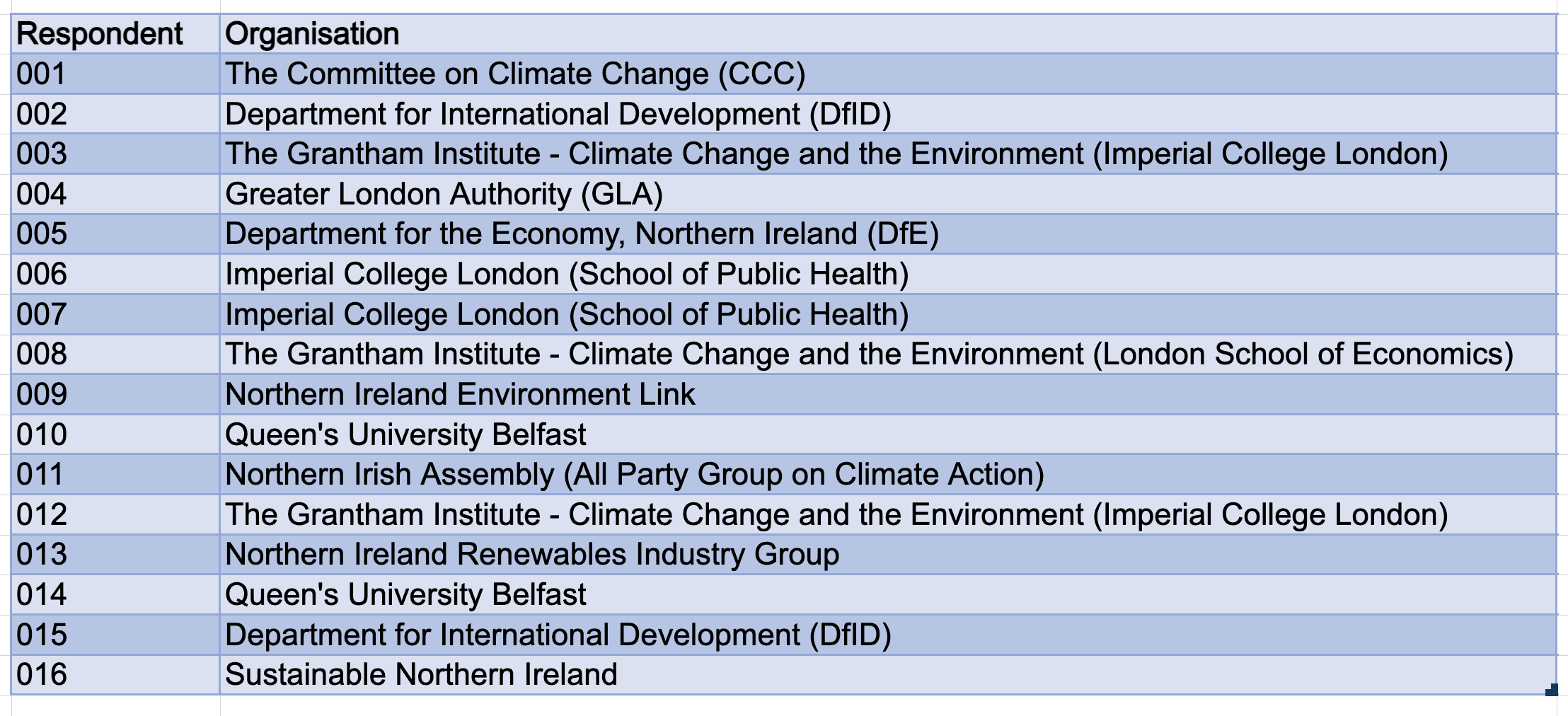

Supplement: Supplementary file 4 — Additional file4 (PNG 395 KB) [file 43621_2021_51_MOESM4_ESM.png]

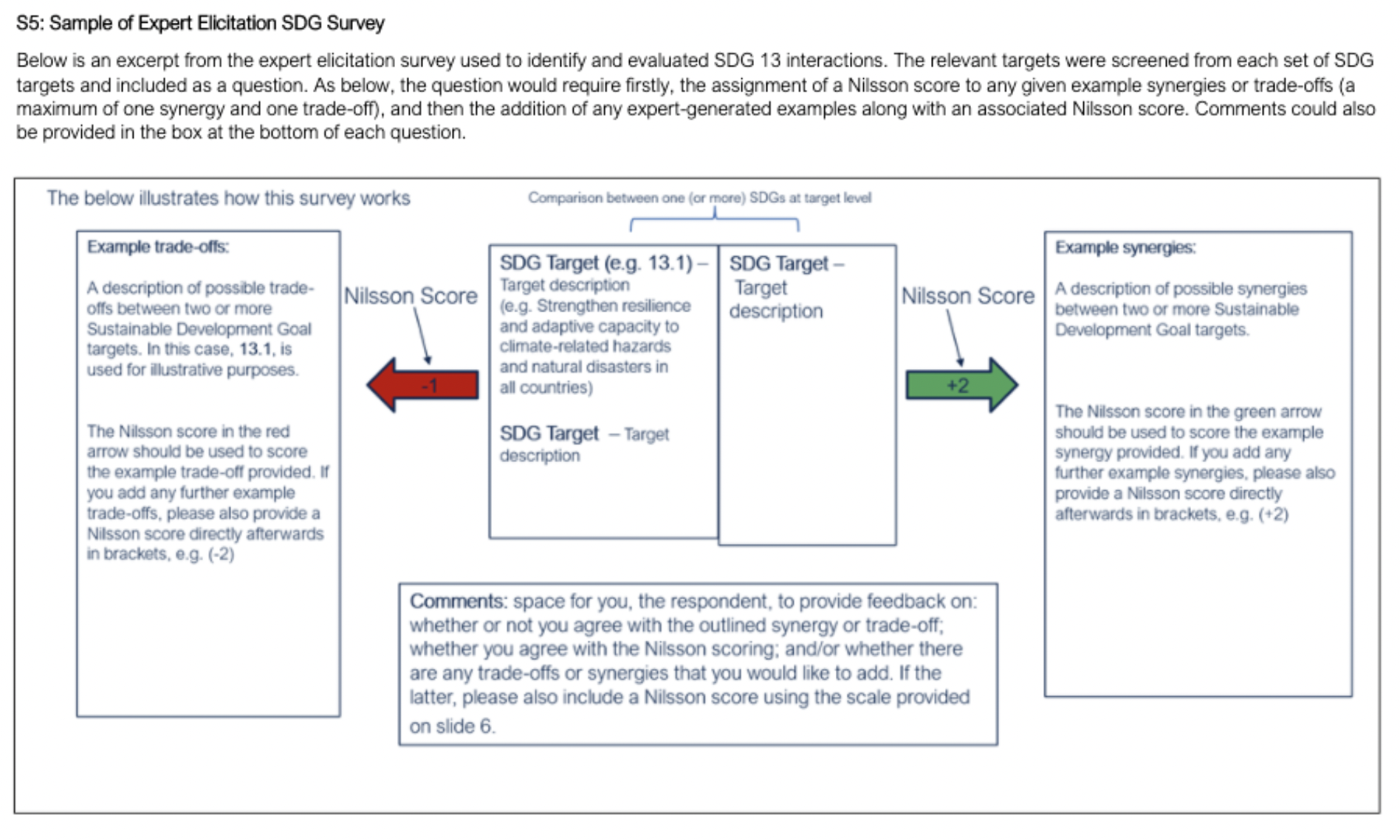

Supplement: Supplementary file 5 — Additional file5 (PNG 1587 KB) [file 43621_2021_51_MOESM5_ESM.png]
