# Supplementary material for: A hybrid approach to identifying and assessing interactions between climate action (SDG13) policies and a range of SDGs in a UK context
Source: Discov Sustain. 2021 Oct 5;2(1):43. doi: 10.1007/s43621-021-00051-w (PMC8491187; doi:10.1007/s43621-021-00051-w)
Supplement: Supplementary file 6 — Additional file6 (PDF 27755 KB) [file 43621_2021_51_MOESM6_ESM.pdf]

S6 – Summary of full responses from expert elicitation phase

SDG Target(s)	SDG Target(s)	Example synergies	Description	Nilsson Score	Average	Example trade-offs	Description	Nilsson Score	Average
13.1 – Streng then resilience and adaptive capacity to climate-related hazards and natural disasters in all countries<br><br>13.2 – Integrate cli mate change measures into national policies, strategies and planning	3.4 – By 2030, reduce by one third premature mortality from non-communicable diseases through prevention and treatment and promote mental health and well-being	By improving the UK's adaptive capacity to climate change and its impacts, and by integrating effective political and social measures to tackle climate change whilst also raising awareness as to its dangers, we can improve the health of citizens.<br>The literature supporting this discusses both the physical and mental impact of climate-related disasters and the psychological stress caused by climate change more broadly	Improved adaptive capacity and climate change mitigation protects physical and mental health of citizens	[+3, +2, +1, +2, +3, +3, +1, +3, +3, +3, +2, +2, +2]	+2.3	Decarbonising electricity and heating risks increased fuel costs, exacerbating fuel poverty and placing a high number of vulnerable people at risk. This is likely to have significant impacts on both the physical and mental well-being of the fuel poor	Decarbonising energy risks increase fuel costs, impacting health of fuel poor.	[-1, -2, -2, -2, -1, -1, 0, -1, -1, -1, 0, -1, -1, -2, -1]	-1.1
						Job losses from carbon-intensive sectors and communities could lead to poor health, long-term sickness, depression.	Job losses from carbon-intensive sectors could lead to chronic stress and depression	-1	-1.0
		By incorporating climate change across the national policy approach, we are likely prioritising active travel whilst	Climate action prioritises active travel and deprioritises private mobility	[+2]	+2.0	Adaptation through regulating house building so as not to be on flood plains could raise housing costs (by reducing	Restricting house building to lower risk areas (e.g.	-1	-1.0

		deprioritising individual, private mobility, creating a positive health impact overall				housing supply), thereby negatively impacting income and health.	away from floodplains) restricts supply and impacts health		
		Nature-based solutions (e.g. afforestation) lead to positive effects on wellbeing and mental health	Nature-based solutions (e.g. afforestation) lead to positive effects on wellbeing and mental health	+2	+2.0	Shift to EVs and banning of ICEs could hamper access for more remote households unless infrastructure built. (vis a vis public transport e.g. buses, trains, flights to UK islands).	Swift to EVs and ICE ban hamper remote household access/connectivity	-1	-1.0
		There will be a buffering effect from improved climate hazard management on food supply/price variability. This will help to stabilize pricing and consumption, ensuring access to nutritious food	Improved climate hazard management stabilizes food prices and supply – benefits on nutrition	+1	+1.0	**Mismanaging the deployment of bioenergy has the capacity to damage human health through environmental pollution (e.g. air and water)**	Mismanaged bioenergy deployment damages human health through environmental pollution	[-1, -2, -1, -1, -1, -1, -1, -2, -1, 0, -1, -2, -1, -1]	-1.1
		More sustainable food systems, improved animal standards, sustainable agriculture	More sustainable agricultural and food technology systems	+1	+1.0	Local pollution also a risk form local mining / production / recycling of extractive resources associated with low-carbon technologies e.g.	Local pollution from mining, production and recycling of low carbon	-1	-1.0
				+2	+2.0				

		Several policies that address climate change also address the prevention of chronic diseases (e.g. active transport prevents diabetes, obesity, CVD; decrease in meat intake prevents CVD and reduces GHG emissions)	Climate policies prevent chronic disease (e.g. active transport prevents diabetes)			Lithium deposit mines, nuclear waste treatment sites	technology materials		
						Increased demand for biofuels and tree planting/carbon sequestration leads to pressure on food supply/prices with effects on health	Increased demand for bioenergy and afforestation places pressure on food prices, which could impact health	-1	-1.0
						There may be as yet unknown impacts of new building designs/retrofitting (e.g. heat stress, poor ventilation, damp etc.)	Unknown impacts of new building design	-1	-1.0
						Carbon tax likely to impact those already on low incomes, who are may already have a disproportional number of health problems	Carbon tax has greatest impact on the poor and their health	-1, -2, -2	-1.7
								-1	-1.0

						Developing climate policies raises profile of issue and can exacerbate climate anxiety (mental health impact)	Communicating climate change can exacerbate climate anxiety		
						There are several ‘fragile’ sectors in the UK that could be adversely impacted by climate policies. E.g. oil and petroleum, mining, or agri-food industry where as the effects of climate change worsen, and as we orientate ourselves post CAP farmers could be pushed into further economic hardship, as well as physical and mental work-related stress.	Climate policies cause sectoral decline and economic hardship	-1	-1.0
						If we insulate buildings but do not design them to also be cool and well ventilated, then this could increase heat stress during the summer months, especially as the climate continues to warm	Building design might focus on winter months at the expense of optimal hot weather design	-1	-1.0
						The expansion of bioenergy in the UK may also have other health-related trade-offs, particularly around limiting access to nature necessary for mental wellbeing and	Bioenergy expansion can restrict access to nature and affect food prices	-1	-1.0

						restricting areas of arable land important for nutrition			
13.3 – Improve education, awareness-raising and human and institutional capacity on climate change mitigation, adaptation, impact reduction and early warning	3.4 – By 2030, reduce by one third premature mortality from non-communicable diseases through prevention and treatment and promote mental health and well-being	Improving our capacity to respond to climate change is likely to ameliorate its deleterious mental and physical impacts as well as raise the profile of climate-change as a political and social issue	Climate policies tackle physical and mental health impacts and promotes socio-political action	[+2, +2, +2, +3, +3, +1, +2, +3, +2, +2, +3, +2]	+2.3	No known trade-offs<br><br>Engaging the public on climate change can create stress and anxiety, so we must message clearly	Communicating climate change can exacerbate climate anxiety	0<br><br>[-1, -2, -2]	-1.7

		Mitigating climate change and its effects will reduce heat stress (heat exhaustion/stroke) in buildings where we live and work	Climate change mitigation will reduce heat stress at work and at home	+1	+1				
**13.1** – Strengthen resilience and adaptive capacity to climate-related hazards and natural disasters in all countries<br><br>**13.2** – Integrate climate change measures into national policies, strategies and planning	**3.6** – By 2020, halve the number of global deaths and injuries from road traffic accidents	**Employing climate policies which encourage alternative, low carbon transport, such as public transport, cycling and walking, is likely to reduce deaths from road traffic accidents**	Low carbon and active transport promotion reduces deaths from road traffic accidents	[+2, 0, +2, +3, +3, +3, +2, +2, +2, +3, +2, +3, +2, +3]	+2.2	**A possible outcome of increased cycling is a rise in cycling deaths, particularly if the increase is not accompanied by sufficient investment in safe infrastructure (e.g. segregated cycle highways)**	Increased cycling could create rise in cycling deaths without necessary infrastructural improvements	[-1, 0, -1. 0, -2, -2, -1, 0, -1, 0, -1, -1, 0, 0, -1]	-0.7
						Other forms of active travel, such as walking may be promoted and these also require infrastructural change alongside public awareness campaigns in order to ensure safety is paramount (urban and rural areas)	Other forms of active travel also require infrastructural change to make them safe	-1	-1.0
						Policies which promote alternative transport and therefore disincentivise cars	Climate-friendly travel solutions likely differed	-1	-1.0

						are likely to be urban-centric, and therefore omit rural areas. As such, the same benefits may not be seen in rural areas.<br><br>Promotion of electric personal vehicles can result in similar trade-offs around RTIs and congestion	for urban and rural areas<br><br>Promotion of electric personal vehicles doesn't solve congestion and RTIs	-1	-1.0
**13.1** – Strengthen resilience and adaptive capacity to climate-related hazards and natural disasters in all countries<br><br>**13.2** – Integrate climate change measures into national policies, strategies and planning	**3.9** – By 2030, substantially reduce the number of deaths and illnesses from hazardous chemicals and air, water and soil pollution and contamination	**Promoting the use of renewable energy reduces reliance on extractive industries and their products (e.g. crude oil), which pollute the natural environment, both pre- and post-combustion**	Investment in renewable energy reduces reliance on extractive industries and therefore environmental pollution	[+3, +1, +3, +3, +2, +3, +3, +3, +2, +2, +2, +2, +3, +2]	+2.5	**Some forms of renewable energy, such as bioenergy, have the capacity to damage environmental and human health if improperly managed**<br><br>Chemicals in low-carbon technologies and involved in their manufacture e.g. Li-ion batteries, solar PV, electrolysers could leak into environment if not well managed	Bioenergy can damage environmental and human health, if mismanaged<br><br>Low carbon technology materials risk polluting environment at both extraction and disposal	[-1, -1, -1, 0, -1, -1, -1, -1, -1, 0, -1, -1, -1]<br><br>[-1, -2]	-0.8<br><br>-1.5

						Adaptation using GM crops could have negative consequences if improperly tested / regulated	GM climate crops could have unintended consequences	-1	-1.0
						If CO_2 from CCS leaks then it could be a powerful pollutant	CO_2 from CCS leaks will be a powerful pollutant	-2	-2.0
						Some forms of geoengineering could have unknown environmental impacts which directly impact human health. E.g. solar radiation management	Some forms of geoengineering could have unknown environmental and human health impacts	-2	-2.0
						Wider environmental impacts (as well as impacts on human labour) of extractive industries and new demands for rare elements for renewables	Wider environmental impacts of renewable technology	-2	-2.0

							material extraction		
**13.1** – Strengthen resilience and adaptive capacity to climate-related hazards and natural disasters in all countries<br><br>**13.2** – Integrate climate change measures into national policies, strategies and planning	**3.3** – By 2030, end the epidemics of AIDS, tuberculosis, malaria and neglected tropical diseases and combat hepatitis, water-borne diseases and other communicable diseases	Taking direct climate action is likely to reduce overall global temperature rise, which can lower communication rates for vector- borne diseases such as malaria<br><br>Inherent in climate action is avoided deforestation, strongly linked to zoonotic diseases like SARS-Cov 1 and Cov 2 (potentially)<br><br>By improving our resilience to the impacts of climate change, we will likely also improve our resilience to public health	Climate action reduces temperature rise, lowering communication rates for vector-borne diseases<br><br>Climate action reduces deforestation which has been linked to zoonotic diseases e.g. SARS-Cov 1 & 2<br><br>Improved resilience to climate change also improves resilience to	[+3, +1, +1, +1, +3, 0, +1, +1, +1, +3, +2]<br><br>+3<br><br>+2	+1.5<br><br>+3<br><br>+2	Policies such as the restoration of wetlands could, if mismanaged, promote the spread of vector-borne diseases in the UK	Afforestation of wetland restoration could promote vector-borne diseases	[-1, -1, -1, -1, 0, -1, 0, 0, -1, -1]	-0.7

		pandemics (improved healthcare, for example)	public health risks (e.g. pandemics)						
13.1 – Strengthen resilience and adaptive capacity to climate-related hazards and natural disasters in all countries<br><br>13.2 – Integrate climate change measures into national policies, strategies and planning	3.D - Strengthen the capacity of all countries, in particular developing countries, for early warning, risk reduction and management of national and global health risks	There is a clear relationship between climate change and public health. This is true both in terms of the physical effects, such as injury or death from flooding, and the mental effects, such as stress and anxiety. Developing appropriate climate mitigation and adaptation policies is therefore an essential component of managing national and global health risks	Climate change policies protect and ameliorate public health (both physical and mental)	[+3, +3, +2, +3, +3, +2, +3, +2, +3, +2, +3, +3, +3]	+2.7	"Law of unintended consequences" i.e. inability to think through all risks and trade-offs could heighten the riskiness. E.g. rapid transition could cause disruption leading to financial sector and real economy downturn and limit capacity to spend on early warning and risk management processes in other sectors.<br><br>There may be detrimental net environmental climate effects from our mitigation efforts (e.g. through unmanaged offshoring)	Rapid transition could cause economic downturn and reduce risk management capacity<br><br>Detrimental net environmental climate effects from mitigation efforts (e.g. offshoring)	-1<br><br>-1	-1.0<br><br>-1.0
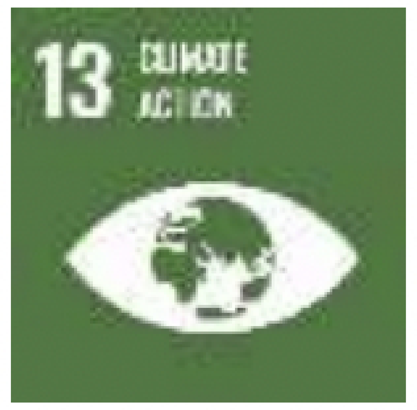	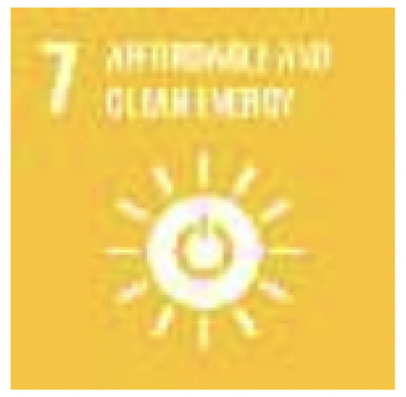								
13.2 – Integrate climate change measures into national policies,	7.1 - By 2030, ensure universal access to affordable, reliable and modern	The pursuit of renewable energy through national policies will significantly expand the roll-out of affordable, reliable and modern energy services, for example by requiring smarter	Renewable energy policies expand roll-out of modern energy services and systems	[+3, +3, +2, +3, +2, +3, +2, +3, +3, +3,	+2.7	It may be that an excessive focus on solely domestic climate action, e.g. renewable energy policies, does less good overall than supporting an equivalent effort in developing countries	Excessive focus on domestic climate action does less good than equivalent international effort	[-1, 0, 0, -1, -1, -1, -1, 0, -1, 0]	-0.6

strategies and planning	energy services<br><br>**7.2** - By 2030, increase substantially the share of renewable energy in the global energy mix	energy generation and demand-side balancing		+1, +3, +3]		UK focus on national decarbonization and adaptation could limit funding for overseas development aid support for off-grid energy access	Focus on UK decarbonisation could limit funding for international decarbonisation	-1	-1.0
		UK innovation in tackling climate change will drive improvements in the availability and price of renewables (+1)	UK innovation drives down price of renewables	+1	+1.0	7.1 alone (i.e. without 7.2 as context) could promote rapid extension and expansion of existing, carbon-intensive electricity grids in emerging economies with poor electricity access	Increasing energy access could come without uptake of renewables	-1	-1.0
						Depending on how implemented, increased costs for energy may generate fuel poverty and disincentivize people accessing energy	Increased energy costs exacerbate fuel poverty and disincentivise energy access	-2	-2.0
**13.2** – Integrate climate change measures into national policies, strategies and planning	**7.3** - By 2030, double the global rate of improvement in energy efficiency	In the context of the current UK grid mix, improving energy efficiency constitutes climate action insofar as it reduces overall energy demand	Improved energy efficiency reduces total energy demand	[+3, +3, +3, +3, +3, +2, +2, +3, +2, +3, +2, +3]	+2.7	No known trade-offs	-	0	0
						We should be mindful of potential non-energy related negative impacts of people increasing consumption in other areas of their life because they know that their energy consumption has reduced	Non-energy impacts of increase consumption (Jevons Paradox)	-1	-1.0

						If we insulate buildings but do not design them to also be cool and well ventilated, then this could increase heat stress during the summer months, especially as the climate continues to warm, creating a need for further cooling which reduces net energy efficiency. The UK has made created this sort of problem before in the past (e.g. early heat networks in buildings)	Unintended consequences of building design	-1	-1.0
13.2 – Integrate climate change measures into national policies, strategies and planning	7.A - By 2030, enhance international cooperation to facilitate access to clean energy research and technology, including renewable energy, energy efficiency and advanced and cleaner fossil-fuel technology, and promote investment in energy infrastructure and clean	Taking decisive climate action both necessitates and facilitates clean energy research, technology and funding. Developing climate policy is likely to act as a stimulus for research in this area	Climate action facilitates clean energy research and vice versa	[+2, +3, +2, +3, +3, +3, +3, +2, +3, +2, +3, +3, +2]	+2.6	Overtly national decarbonization focus risks limiting international cooperation (-1)<br><br>It is important that RD&D is funded across the full spectrum of technology costs, so as to prevent exacerbating socio-economic inequalities, or the monopolisation of climate critical technologies amongst a few large actors.	Excessive domestic decarbonisation limits international cooperation<br><br>R&D focus too narrow	-1<br><br>-1	-1.0<br><br>-1.0

	energy technology					Ownership and control of patents of these technologies should be shared equitably, rather than used to monopolise renewable energy technologies/technology access	Monopolisation of renewable energy technologies, e.g. patent ownership	-1	-1.0
						Spending on RD&D could divert money from implementing known solutions	RD&D spending diverts money from ready-to-deploy solutions	-1	-1.0
						Innovation doesn't just happen in clean technologies, but also for fossil fuels, e.g. UK shale in recent years. Target 7A includes fossil fuel R&D and therefore poses a potential trade-off because it doesn't focus on clean energy which we know we need to focus on in the long-term	Fossil fuel R&D contributes to this target	-1	-1.0
13.2 – Integrate climate change measures into national policies, strategies	7.B - By 2030, expand infrastructure and upgrade technology for supplying modern and sustainable	A key aspect of climate action policy is supporting the upgrade and expansion of sustainable energy and transport infrastructure. Lessons learned domestically in developed countries can be	Domestic upgrade and expansion of low carbon infrastructure can support international development	[+3, +3, +3, +2, +3, +3, +2, +2, +3, +2, +3, +3, +2]	+2.6	Potential conflict with national vs international obligations/ strategies	Potential trade-off between national goals versus international obligation	-1	-1.0

and planning	energy services for all in developing countries, in particular least develop ed countries, small island developing States, and land-locked developing countries, in accordance with their respective pr ogrammes of support	exported to those which are still developing				There is a risk that the wrong/obsolete tech is passed to developing countries (as was seen with some agricultural and pesticide technology in the past). We must make sure that the transfer of knowledge, resources and support is transparent and in line with recipient priorities.<br><br>There is a risk that the Global North determines those technologies and solutions suitable for the Global South, rather than least developed nations deciding for themselves what works for them<br><br>Potential for new technology to be promoted at expense of existing sustainable solutions - solutions that are new are not necessarily the most appropriate	Risk of unsuitable/obsolete technology being passed to developing countries<br><br>Paternalistic and non-inclusive solutions for developing countries<br><br>Newest solution not necessarily most appropriate	-1<br><br>-1<br><br>-1	-1.0<br><br>-1.0<br><br>-1.0
13 CLIMATE ACTION	8 DECENT WORK AND ECONOMIC GROWTH								

**13.2** – Integrate climate change measures into national policies, strategies and planning	**8.4 -** Improve progressively, through 2030, global resource efficiency in consumption and production and endeavour to decouple economic growth from environmental degradation, in accordance with the 10-year framework of programmes on sustainable consumption and production, with developed countries taking the lead	Policies which promote climate action are likely to improve global resource efficiency in consumption and production, e.g. the use of bioenergy systems which reduce food waste whilst generating low carbon heat and power<br><br>Lifestyle changes, the increased deployment of renewable energy systems and improvements in energy efficiency will lower resource demand as well as rates of environmental degradation<br><br>There are also productivity benefits which come as a result	Climate action improves resource efficiency, e.g. Anaerobic Digestion from food waste<br><br>Lifestyle changes, increased renewables deployment and energy efficiency will lower resource demand<br><br>Productivity benefits of	[+3, +2, +2, +3, 0, +3, +2, +2, +2, +2, +3, +2, +3, +2]<br><br>[+1, +2, +2, +3, +2, +2, +3, +1, +3, +2, +2, +2]<br><br>+2	+2.2<br><br>+2.1<br><br>+2.0	No known trade-offs<br><br>Decarbonising certain industries is likely to have considerable impacts in terms of unemployment. Where no alternative, state-funded retraining is provided, there is a risk that such climate action works against job security and economic growth.<br><br>There is a risk that measures to decouple economic growth and emissions in the UK, without due accounting for supply chain emissions (as currently under discussed at UNFCCC, for example) will simply offshore emissions to other countries	-<br><br>Failure to achieve a just transition stifles economic growth and security<br><br>Measures decoupling economic growth and emissions could lead to offshoring	0<br><br>-1<br><br>-2	0<br><br>-1.0<br><br>-2.0

		of (energy) efficiency improvements	energy efficiency improvements			It may be that we cannot achieve the necessary degree of climate action whilst pursuing current rates of economic growth	Current rates of economic growth and climate action are in tension	-1	-1.0
						Economic growth, especially in the UK agricultural sector is a significant driver of environmental degradation	Inverse correlation risk (e.g. growth of agricultural sector drives environmental degradation	-1	-1.0
						The Jevons paradox/rebound effect – even as we become more efficient in our technologies and reduce energy demand, there may be some unforeseen impact of no longer constrained consumption	Poorly understood non-energy impacts (rebound effect)	-1	-1.0
						This comparison produces an ideological trade-off insofar as measurable progress towards 8.4 signifies success on a very orthodox conception of environmental economics – that is, the notion that economic growth and environmental degradation can in fact be decoupled. Prospective policy should challenge this view to find the	Assumes economic growth and environmental degradation can be coupled under prevailing economic model	-1	-1.0

						best solutions for a more sustainable future world.<br><br>It's not clear that, the more efficiently we use resources the lower consumption rates will be. In fact, if improved efficiency = economic growth then consumption is likely to increase. This must be managed by intelligent policy design and behavioural shifts.	Improved efficiency may actually increase growth and consumption	[-1, -2]	-1.5
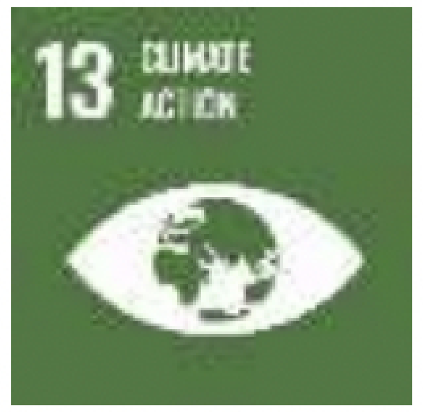	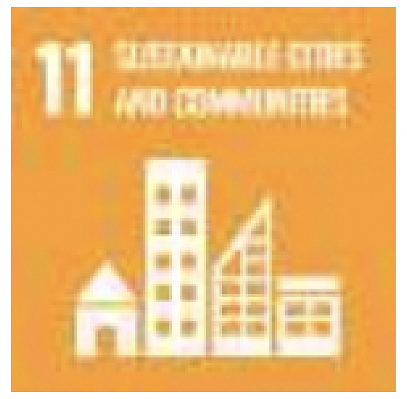								
13.2 – Integrate climate change measures into national policies, strategies and planning	11.2 - By 2030, provide access to safe, affordable, accessible and sustainable transport systems for all, improving road safety, notably by	Integrating climate change measures into national policy approaches includes rolling out decarbonised transp ort systems which improve safety and, in some cases, accessibility too. The decarbonisation of transport should be seen as an opportunity to significantly improve its quality and accessibility	Decarbonisation of transport as opportunity to improve accessibility	[+3, +2, +3, +2, +2, +1, +3, +2, +3, +2, +1]	+2.2	There is a risk that restricting private mobility and failing to make suitable accessibility provisions in the design of new shared transport systems will negatively impact the most vulnerable in society: women, children, the elderly and disabled<br><br>If public transport costs are not managed, then access will	Curtailment of private mobility and poorly designed public transport impacts vulnerable groups<br><br>If cost of decarbonising transport is transferred to users, this will	[-2, -1, -2, -1, -2, -1, -1, -2, -1]<br><br>[-1, -1]	-1.4<br><br>-1.0

	expanding public transport, with special attention to the needs of those in vulnerable situations, women, children, persons with disabilities and older persons					track existing socio-economic inequalities	track existing socio-economic inequalities		
13.2 – Integrate climate change measures into national policies, strategies and planning	11.3 - By 2030, enhance inclusive and sustainable urbanization and capacity for participatory, integrated and sustainable human settlement planning and management in all countries	Sustainable urbanisation can be achieved through a number of policies which also take direct climate action. For example, low carbon transport systems or high energy efficiency retrofit and new-build programmes	Climate action through sustainable urbanisation (e.g. high energy efficiency retrofits)	[+3, +2, +2, +2, +3, +1, +2, +2, +3, +2, +3, +3, +2, +2]	+2.3	No known trade-off	-		
						There is a risk that the pursuit of sustainable urbanisation could be carried out by only a few, powerful actors, and now involve sufficient participation from the full spectrum of stakeholders in an accessible, participatory and democratic fashion.	Risk of unequal and non-democratic decision making processes which perpetuate existing inequalities	[-1, -1]	-1.0
						Risk that local participatory urban planning can neglect wider considerations outside of the settlement, e.g. at the regional or national level. Settlements do not exist in a resource/energy vacuum.	Local decision making processes can neglect impact outside of settlement (e.g. urban versus rural)	-1	-1.0
							Participatory democracy can	-1	-1.0

						Participatory democracy and complicated bureaucratic decision-making processes can significantly slow progress	slow urgent climate action		
13.2 – Integrate climate change measures into national policies, strategies and planning	11.4 - Strengthen efforts to protect and safeguard the world's cultural and natural heritage	Indicators for this goal include the conservation of natural capital, which aligns with the environmental protectionism afforded by climate action policies. Examples include peatland restoration and afforestation	Climate action includes conservation of natural capital	[+3, +1, +3, +2, +3, +3, +1, +3, +2, +3, +2, +3, +2, +2]	+2.4	Massive expansion of low energy density renewables might impinge on natural resources like farmland, national parks, forests and so on.	Unmanaged expansion of low carbon technology likely to impinge on sites of natural heritage (e.g. HS2)	-1	-1.0
		Equally, by mitigating certain impacts of climatic change, e.g. flooding, we can protect cultural and natural heritage sites in UK (particularly coastal and fluvial areas)	Mitigating climate event impacts (e.g. flooding) can protect sites of cultural and natural heritage/ importance	+2	+2.0	Decarbonising/descaling high carbon sectors such as agriculture may be a form of cultural erosion in terms of rural heritage	Decarbonising/scaling-down high carbon sectors may cause cultural erosion	[-1, -1, -1]	-1.0
						We must consider the social (and economic) effects of	Socio-cultural impacts of conservation	[-1, -1]	-1.0

						climate policies which, for example, seek to restore or extend areas of conservation, as there are those whose livelihoods depend on resources from these same areas. We must not make changes to their ability to access these resources/areas without first considering alternatives/impacts<br><br>There is a risk that even purportedly climate positive policies (e.g. HS2) can rob individuals and communities of their connection to nature, eroding natural and cultural heritage.<br><br>Older, protected buildings are much more difficult to make energy efficient/retrofit (both practically and bureaucratically). To improve energy efficiency of older housing stock, we may need to make some difficult trade-offs regarding the preservation of cultural or historical value	Climate positive policies can rob individual and communities of connection to natural heritage<br><br>Older, protected building harder to retrofit	[-1, -1]<br><br>-1	-1.0<br><br>-1.0
13.2 – Integrate climate change	11.5 - By 2030, significantly reduce the	Tackling climate change through appropriately designed policy will reduce the risk and associated harm of those	Climate mitigation and adaptation policies will reduce harm and	[+3, +2, +3, +2, +2, +3, +2, +3,	+2.7	No known trade-offs	-	0<br><br>-2, -1	0<br><br>-1.5

measures into national policies, strategies and planning	number of deaths and the number of people affected and substantially decrease the direct economic losses relative to global gross domestic product caused by disasters, including water-related disasters, with a focus on protecting the poor and people in vulnerable situations	impacted by climate-related disasters	death from climate-related disasters	+3, +3, +3, +3, +3]		There is a risk that focusing exclusively on climate-related disasters means that other shocks (e.g. pandemics) are ignored/not sufficiently mitigated against, which may affect incomes/deaths etc. – and which if you have recurrent shocks, will undermine resilience to climate-related disasters and our performance on climate targets.<br><br>One trade-off could be the relative cost of adaptation compared to the cost that the disaster would have imposed – who pays for that adaptation and how do we ensure the costs do not fall disproportionately on the poorest in society?	Excessive focus on climate-related disasters neglects protective measures from other shocks<br><br>Cost of adaptation versus cost of disasters	-1	-1.0
13.2 – Integrate climate change measures into national policies, strategies and planning	11.6 - By 2030, reduce the adverse per capita environmental impact of cities, including by paying special attention to air quality and municipal and	By improving areas such as energy efficiency and waste management, climate-oriented policies have the capacity to reduce the adverse per capita environmental impact of cities	Climate policies reduce per capital environmental impact of cities	[+3, +2, +3, +2, +3, +2, +2, +3, +3, +3, +2, +2]	+2.5	No known trade-offs<br><br>Broader footprint of cities increases generating negative environmental impacts, including energy	-<br><br>Negative environmental impact from growing city footprints<br><br>Risk of	0<br><br>-2<br><br>-1	0<br><br>-2.0<br><br>-1.0

	other waste management	Improving waste would liberate funds for local councils, in that as much as 1/3 of council budgets can be spent on waste management alone	Tackling waste effectively would liberate local authority budgets for climate action	+3	+3.0	Energy from waste might be an ostensibly climate positive policy, but it can incentivise (and monetise) waste streams. Such policies are therefore fundamentally opposed to the concept of zero waste	incentivising waste (e.g. Energy from Waste contracts)		
						Potential risk here of overlooking issues in rural areas, including infrastructure (e.g. public transport) and agriculture (e.g. waste and water pollution)	Urban-centric policies overlook rural needs	-1	-1.0
						We should be mindful of the export of environmental impact from urban to rural areas, e.g. waste management	Risk of exporting environmental impact from cities to rural areas	-1	-1.0
